# Two-stage multi-objective framework for optimal operation of modern distribution network considering demand response program

**DOI:** 10.1038/s41598-024-83284-9

**Published:** 2025-01-06

**Authors:** Mohamed R. Elshenawy, Abdalla Mohamed, A. A. Ali, Magdi A. Mosa

**Affiliations:** Electrical Power and Machines Engineering Department, Faculty of Engineering, Helwan University, Cairo, Egypt

**Keywords:** Demand response program (DRP), Energy management (EM), Incentive rate, Technique for order of preference by similarity to ideal solution (TOPSIS), Elephant herding optimization (EHO), Distribution network operator (DNO), Engineering, Electrical and electronic engineering

## Abstract

To improve the inadequate reliability of the grid that has led to a worsening energy crisis and environmental issues, comprehensive research on new clean renewable energy and efficient, cost-effective, and eco-friendly energy management technologies is essential. This requires the creation of advanced energy management systems to enhance system reliability and optimize efficiency. Demand-side energy management systems are a superior solution for multiple reasons. Firstly, they empower consumers to actively oversee and regulate their energy consumption, resulting in substantial cost savings by minimizing usage during peak hours and enhancing overall efficiency. The Demand Response Program (DRP) and optimal power sharing have gained significant attention to provide technical and economic benefits, while they require an efficient operation framework. Therefore, a two-stage framework is proposed for multi-objective operation of a distribution network with several generation resources. The first stage applies DRP to maximize the distribution network operator’s (DNO) profit by optimizing common incentive rate for all consumers participate in DRP and an individual curtailed power for each consumer. In addition to an individual incentive rate for each consumer participates in DRP which is a new solution in the field of demand side management. The second stage achieves optimal power sharing among generation resources, while considering multiple objectives and incorporating the modified load of the first stage. The multi-objective problem is formulated to reduce energy losses, voltage deviation, total operational cost, gas emissions, and maximize the voltage stability index. The problem is optimized using a combination of the technique for order of preference by similarity to ideal solution (TOPSIS) and the elephant herding optimization (EHO) technique. The framework is validated using a modified IEEE 33-bus that incorporates photovoltaic system, diesel generators, and wind generation system. The proposed framework based on an individual incentive rate DRP provides superior response compared to common incentive rate DRP which reduces the total energy losses by 38.13%, reduces the total generation cost by 9.468%, and reduces the emission by 5.9%.

## Introduction

Energy resources management is crucial globally to provide the necessary power in an efficient and cost-effective manner, as load demand rises due to industrial, economic, and social advances. Distribution networks (DNs) are the most important part in power system for consumer’s connection. The majority of DNs are in radial configuration because they are inexpensive and easy to build, design, and safeguard. However, the disadvantages of radial distribution network are a poor voltage profile and high losses which account most of power losses in the network^1^. Distributed Generation Resources (DGRs) connected to DNs and reconfiguration of DNs. Also, applying Demand Side Management (DSM) are effective methods which improve the performance of DNs, increase the system reliability. Also, minimize the system power losses, minimize the total operational cost, and enhance the voltage profile.

Energy Management System (EMS) manages the sharing power between the grid and Distributed Generation Resources (DGRs) by monitoring, analyzing, and forecasting of power generation of DGRs, load consumption and energy market prices. EMS is optimizing the operation of the network considering technical and economic benefits^2^. DSM is participating in improving performance of the network by managing loads consumption. DSM operations is based on the planning and monitoring of consumer activities. Also, distributing and transmitting the consumed energy along the day to decrease the peak load and carbon emission^3^. Energy efficiency, demand response and strategic load growth are the main categories of DSM. Demand response program (DRP) is the most popular type of DSM. DRP is the program which has been designed to provide the consumer with an opportunity of participating in improving the operation of the network. DRP is decreasing in load consumption or shifting the energy usage during peak periods based on price or incentive payment program^4^.

Author in^5^ applied a developed Mixed-Integer-Linear programming framework for the energy management of the network considering variations in DGRs generation output and the load demand. Also, the energy price. In^6^, a microgrid energy management problem to minimize the operational cost, enhance the voltage at every bus, and maximize the voltage stability index has been solved using equilibrium optimizer (EO). In^7^, two layers EMS, an ant lion optimizer algorithm combined with a fuzzy logic system acting as the primary controller has been designed to solve a multi-objective optimization problem aimed to reduce the operating costs, gasses emission, and power losses. In^8^, the overall generating and operating costs have been reduced using an analytical target cascading theory (ATC). In^9^, an economical dispatching strategy using distributed energy storage in an electrical distribution system comprises can postpone substation expansion to improve the daily operation through reduction of the losses and the income due to peak shifting. In^10^, Golden Jackal Optimization for an energy management of distributed generation resources (DGRs) has been used to reduce the operational cost. In^11^, Fmincon method has been used to optimize operation of DGRs connected to grid in a novel way that maximizes social and political advantages while minimizing emission, costs, and the monetary value of energy acquired from the grid. In^12^, a bi-level stochastic formulation has been solved by mathematical program with complementarity constraints (MPCC) to reduce the operational cost. In order to minimize operating costs, minimize energy losses, and improve voltage profiles considering renewable distributed generators and energy storage, a linearized multi-objective framework for distribution network has been presented in^13^. In^14^, an optimal load flow control strategy of tie link has been proposed for the multiple-microgrid system for minimum cost operation. In^15^, two level multi-objective optimization technique has been illustrated using method based on self-adaptive genetic algorithm (SAGA) and non-linear programming for reducing the power losses and generation cost, gasses emission cost, and enhancing the voltage bus for grid connected distribution network. In^16^, the epsilon method with fuzzy strategy have been used to minimize the operation cost and voltage deviation for microgrid connected to utility grid. In^17^, the optimal Pareto solutions have been obtained using multi-objective decision-making (MODM) framework for managing the energy of a grid connected microgrid to reduce the power not supplied (PNS), the cost of maintenance and operation, and maximization of the reserved energy storage. In^18^, Authors have discussed the EMS considering multi-objectives, where Branch and Reduce Optimization Navigator (BARON) algorithm has been utilized for minimum cost operation scenario, minimum emission operation scenario, and multi-objective scenario. A day-ahead EMS incorporates DRP based on load shifting to reduce the operational cost and increase the reliability of a microgrid considering multiple DGRs and the electrical energy storage system has been presented in^19^. EMS framework has been applied considering load management strategy for price-based and incentive-based DRP in^20^. In^21^, energy management for distribution network to reduce total energy losses, to reduce operational cost, and to maximize voltage stability index maximization considering interruptible service based DRP has been implemented using PSO. An Energy management strategy using confidence-based velocity-controlled particle swarm optimization (CVCPSO) combined with a fuzzy-clustering technique for a grid connected to microgrid considering the DRP has been illustrated in^22^.

Authors in^23–29^ have summarized the optimal EMS to the network considering DRP and variable operating conditions including demand load, solar irradiance, and wind speed variations. In^23^, the non-sorted genetic algorithm (NSGA-II) with TOPSIS has been proposed to solve a multi-objective problem involving DRP based time of use (TOU). In^24^, probabilistic optimal generation multi microgrids with TOU-DRP and RTP-DRP has been done using PSO for minimum operation and emission cost. An optimal EMS strategy has been introduced for multi-interconnected micro-grids considering uncertainty using chance constrained Model Predictive Control (CCMPC) in^25,26^. Author in^27^ have optimized the curtailed power and the Incentive rate of consumers in DRP by Microgrid operator (MGO) using PSO. In^28^, the microgrid’s energy management has been applied while incorporating the DRP in a reconfigured distribution network considering uncertainty in renewable energy resources. Authors in^29^ have suggested a three-stage framework of an energy management to minimize the operational costs and emissions. Also, the peak-to-average ratio (PAR) using CPLEX solver and the max-min fuzzy method considering DRP in conjunction with the uncertainties of load demand and DGRs. In^30^ An approach which presents benefits of PSO and slap swarm optimization (SSO) to find a solution for the optimal operation considering different objectives such as emission limitation, generation cost reduction, voltage enhancement, minimize losses and maximizing voltage stability. Considering the uncertainties for more technical benefits about optimal operation of power network a Non-dominated sorting genetic algorithm-II (NSGA-II) TOPSIS have been presented in^31^. Demand response optimization model combined with an economic dispatch which uses DGRs and load management for an optimal operation that minimizes cost of generation and maximizing DGRs penetration which is illustrated in^32^. The study in^33^ proposed a new solution to design parallel schedule of battery storage with electric vehicle considering the DRP to reduce the total operational cost. Authors in^34^ applied the price based DRP to minimize the operational cost by shifting the peak load to off peak hours. Also, minimize total losses and gas emissions by decreasing the received power from grid. In^35^ the energy management system optimized the operation of electric vehicles with the renewable energy systems to minimize the gas emission and air pollution considering the DRP. The studies in^36,37^ presented an advanced framework of DRP with system energy management considering Hybrid operation of DRP which is based on consumer Incentive rate to reduce the peak load and ensure the reliability of the system. The author in^38^ presents Lyapunov function method to solve only the dynamic economic dispatch problem considering the environmental objective without any technical benefits to a hybrid microgrid network using a distributed optimization algorithm. The study in^39^ presents multi-objectives particle swarm optimization (MOPSO) technique considering the loading uncertainty and shortage in refined oil. Also, the limited supply to improve the operation of the electrical system. Authors in^40^ proposes an optimization technique which maximizing the contribution of new energy sources like photovoltaic systems and wind turbines. Also, hydraulic power sources which have a lack in inertia support and may lead to instability in the system frequency but the proposed optimization technique will maximize the new resource shared power considering the frequency constraints and demand load variations leading to minimize the total operational cost and system operation frequency using a mixed-integer linear programming (MILP) model involves a commercial solvers such as GUROBI or CPLEX. Whereas, the study in^41^ presents a review about methods of controlling in the operation of microgrid to improve its performance. The authors in this review recommend using of decentralized controllers as the best control method in the operation of microgrid comparing to centralized controllers due to decentralized nature structure of microgrid. The review presents the advantages and disadvantages of various methods belong to decentralized controllers such as their flexibility, reliability and the cost of controller installation. Also, the disturbance may be caused by the controller method. In addition to the speed of controller response. A three-stage multi energy trading strategy for a gas-electricity integrated energy system (IES) has been presented in^42^ to solve the multi-energy imbalance problem among energy hubs (EHs) based on the peer-to-peer (P2P) trading mode between the seller and buyer agents determining the optimal energy trading price using multi-bilateral negotiations based on the Raiffa-Kalai-Smorodinsky bargaining solution (RBS). The study in^43^ constructs a communication network to support the operation of Demand Response Program (DRP). Where, the performance of DRP depend on the quality of communication between the control center and the consumers. Taguchi loss function is presented to determine the energy price referring to the total consumer curtailed power. Table 1 illustrates a comparative summary of literature reported works and proposed framework.

**Table 1:** Comparative summary of the literature review and the proposed framework.

Refs.	Algorithm	Single objective	Multi objective	Operation costs	power losses	voltage profile	Emission	VSI	DNO profit	DRP	Daily operation profile
^10^	GJO	✓	**-**	✓	**-**	**-**	**-**	**-**	**-**	**-**	**-**
^12^	MPCC	✓	**-**	✓	**-**	**-**	**-**	**-**	**-**	**-**	**-**
^15^	SAGA	**-**	✓	**-**	✓	✓	**-**	**-**	**-**	**-**	✓
^18^	BARON	**-**	✓	✓	**-**	**-**	✓	**-**	**-**	**-**	✓
^19^	PSO	✓	**-**	✓	**-**	**-**	**-**	**-**	**-**	✓	✓
^20^	BWO	✓	**-**	✓	**-**	**-**	**-**	**-**	**-**	✓	✓
^21^	PSO	**-**	✓	✓	✓	**-**	**-**	✓		✓	**-**
^22^	CVCPSO	✓	**-**	✓	**-**	**-**	**-**	**-**	**-**	✓	✓
^23^	NSGA-II	**-**	✓	✓	✓	**-**	**-**	**-**	**-**	✓	✓
^24^	PSO	✓	**-**	✓	**-**	**-**	**-**	**-**	**-**	✓	✓
^25^	CCMPC	✓	**-**	✓	**-**	**-**	**-**	**-**	**-**	**-**	✓
^27^	PSO	**-**	✓	✓	**-**	**-**	**-**		✓	✓	✓
^38^	Lyapunov function	**-**	✓	✓	**-**	**-**	✓	**-**	**-**	**-**	**-**
^39^	MOPSO	**-**	✓	✓	**-**	**-**	**-**	**-**	**-**	**-**	✓
^40^	MILP [CPLEX solver]	**-**	✓	✓	**-**	**-**	**-**	**-**	**-**	**-**	✓
^42^	RBS	✓	**-**	✓	**-**	**-**	**-**	**-**	**-**	**-**	**-**
^43^	Taguchi loss function	✓	**-**	✓	**-**	**-**	**-**	**-**	**-**	✓	**-**
Proposed strategy	TOPSIS + EHO	**-**	✓	✓	✓	✓	✓	✓	✓	✓	✓

This article proposes an efficient multi-objective-two-stage framework for the operation of distribution network comprises distributed generations. The framework employs both the demand response program-based incentive rate and energy management technique to enhance the profit of distribution network operator (DNO), decrease the network losses, enhance stability of the voltage, minimize the generation cost, and reduces the greenhouse gas emissions. The first stage optimizes the profit of DNO by implementing common and individual incentive rates based DRP for a group of consumers with different degrees of discomfort toward power curtailment. The second stage utilizes the presence of different generation resources to optimize the system operation subjected to modified load of the first stage. The presented framework is applied on the 33-bus radial distribution network that includes dispatchable DGs (such as conventional generators) and renewable based DGRs (such as solar or wind). The multi-objective problem is solved by employing the hybrid algorithm comprises the Technique for Order of Preference by Similarity to Ideal Solution (TOPSIS) and the Elephant Herding Optimization (EHO). The illustrated model has been applied using MATLAB software.

Although the richness of the literature. But the proposed work presents an advanced solution in the field of energy management and demand side management. Where, Elephant Herding Optimization (EHO) technique is combined with TOPSIS approach for multi-objectives energy management considering the technical and economic benefits in addition to the environmental benefits throughout the day. Also, EHO technique is applied on DRP to optimize an individual incentive rate for each consumer participate in DRP in contrast most existing works which optimize a common incentive rate for all consumers participate in DRP which may causes more discomfortability and unsatisfaction about some consumers who participate in DRP. So, an individual incentive rate in this research presents a new solution for more flexible operation of DRP application and presents more satisfaction about consumers which will encourage them for more participation in DRP.

The following points illustrate the contribution of this work:

1. Bi-Level framework is introduced to optimize the operation of the distribution network connected to DGRs and the utility grid. The first level maximizes DNO profit by implementing the DRP and optimize the consumer incentive rate and power curtailment. The second level is multi-objective optimal power sharing between DGRs and the grid.
2. Investigating curtailed power optimization for every consumer participating in DRP, considering common and individual consumer incentive rates as two different case studies for limiting the total energy consumption and maximizing the DNO profit and enhancing the system performance.
3. Individual incentive rate (IIR) for each consumer participates in DRP regards as a new solution which introduced in this paper to optimize the curtailed power for every consumer based on their demand load and inability factor achieving more flexibility and comfortability to consumers participate in DRP.
4. Multi-objective optimal power sharing between DGRs and the grid is performed while considering several technical, economic and environmental benefits, including minimizing total energy loss, maximizing the voltage stability index, reducing the generation cost, minimizing the total voltage deviation, and limiting harmful gas emissions.

The remainder of the paper is designed as follows: “Radial distribution system with distributed generation” section presents the proposed distribution network and provides a detailed description of its characteristics. “Proposed operational framework” section describes the proposed framework for demand response program and optimal power sharing for formulation of the Multi-Objective Problem. “Modelling of the operational framework” section illustrates the proposed technique of elephant herding optimization and TOPSIS approach for multi-objective problem optimization. “Proposed strategy” section analysis the implementation of different incentive rate strategies for DRP. “Modelling of the operational framework” section investigates the obtained results of the optimal power sharing with different DRP strategies. “Conclusion” section concludes the paper by summarizing main contributions of the research and highlighting benefits of the proposed framework.

## Radial distribution system with distributed generation

**Fig. 1:**
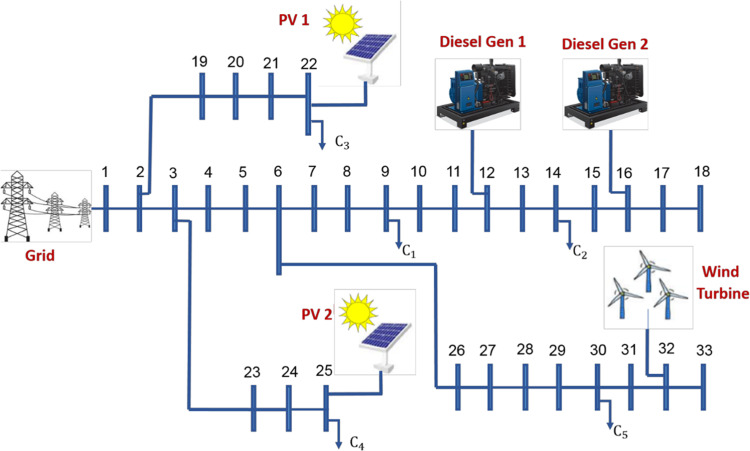
33-Bus radial distribution network connected to DGRs.

**Fig. 2:**
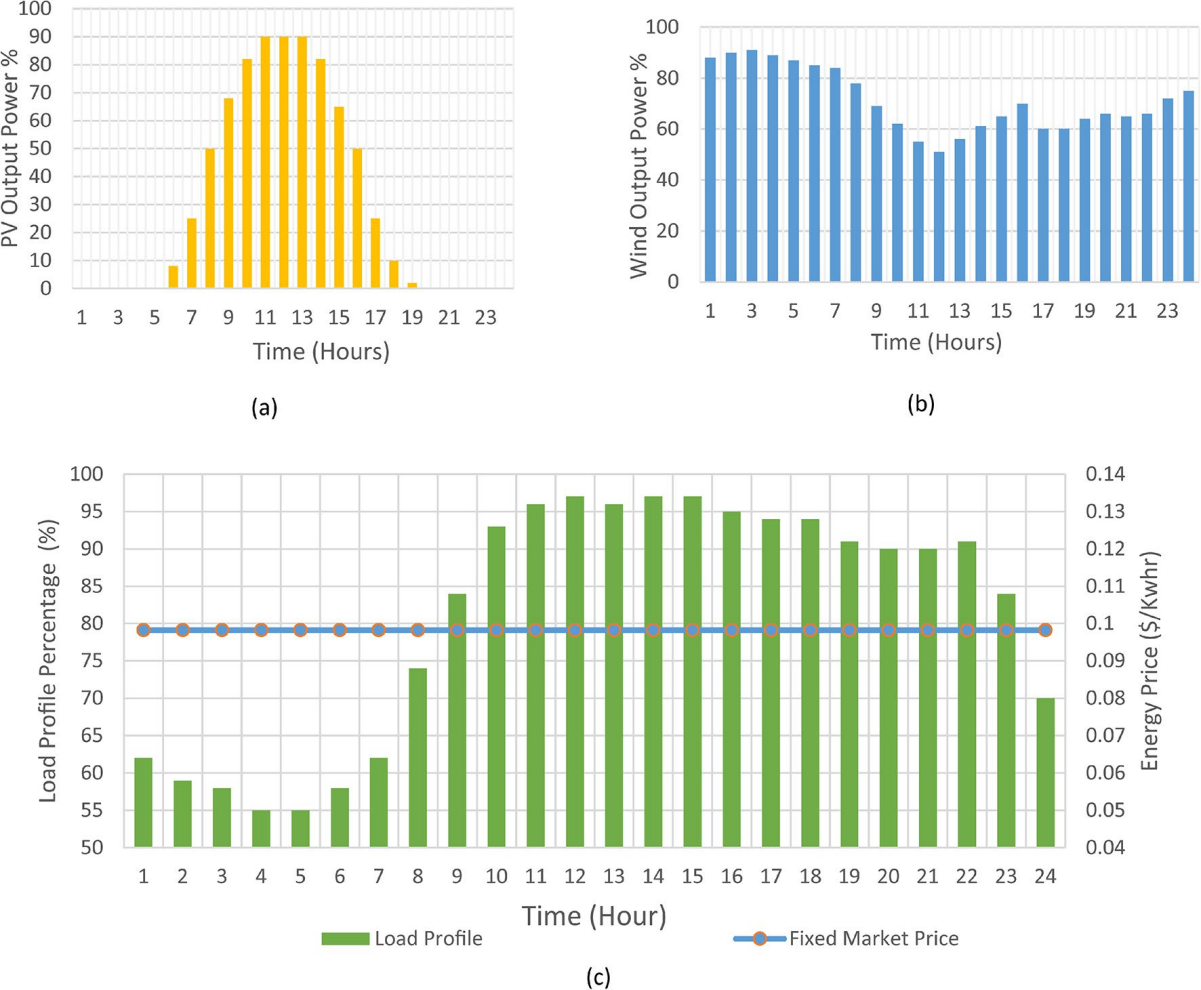
(**a**) Daily percentage of PV generation, (**b**) Daily percentage of Wind generation and (**c**) Daily Load profile with fixed market Price.

IEEE 33-bus radial distribution network is implemented as an example to ensure the validation of the proposed framework. Distribution network operates at a nominal voltage of 12.66 kV and has a base power of 10 MVA, with a nominal demand load of 3.715 MW and 2.3 Mvar for real and reactive power respectively^44,45^. Within the proposed model as illustrated in Fig. 1, there are number of five distributed generation resources (DGRs) connected to distribution network. Two diesel generators (each with a capacity of 0.5 MVA) located at bus 12 and bus 16. Additionally, there are two photovoltaic (PV) systems with capacities of 0.478 MVA and 0.425 MVA, located at bus 22 and bus 25, respectively. Furthermore, a wind generator with a capacity of 0.5 MVA is connected to bus 32. There are only five consumers (C1 to C5 connected to buses {9, 14, 22, 25, and 30}) are assumed participate in the demand response program. Figure 2 illustrates the per unit load profile at all buses before being involved in the DRP and the fixed energy price throughout the day, as well as the per unit variations in generation power from wind turbines and photovoltaic systems throughout the day. Also, the proposed framework can be applied on various models with different characteristics.

## Proposed operational framework

**Fig. 3:**
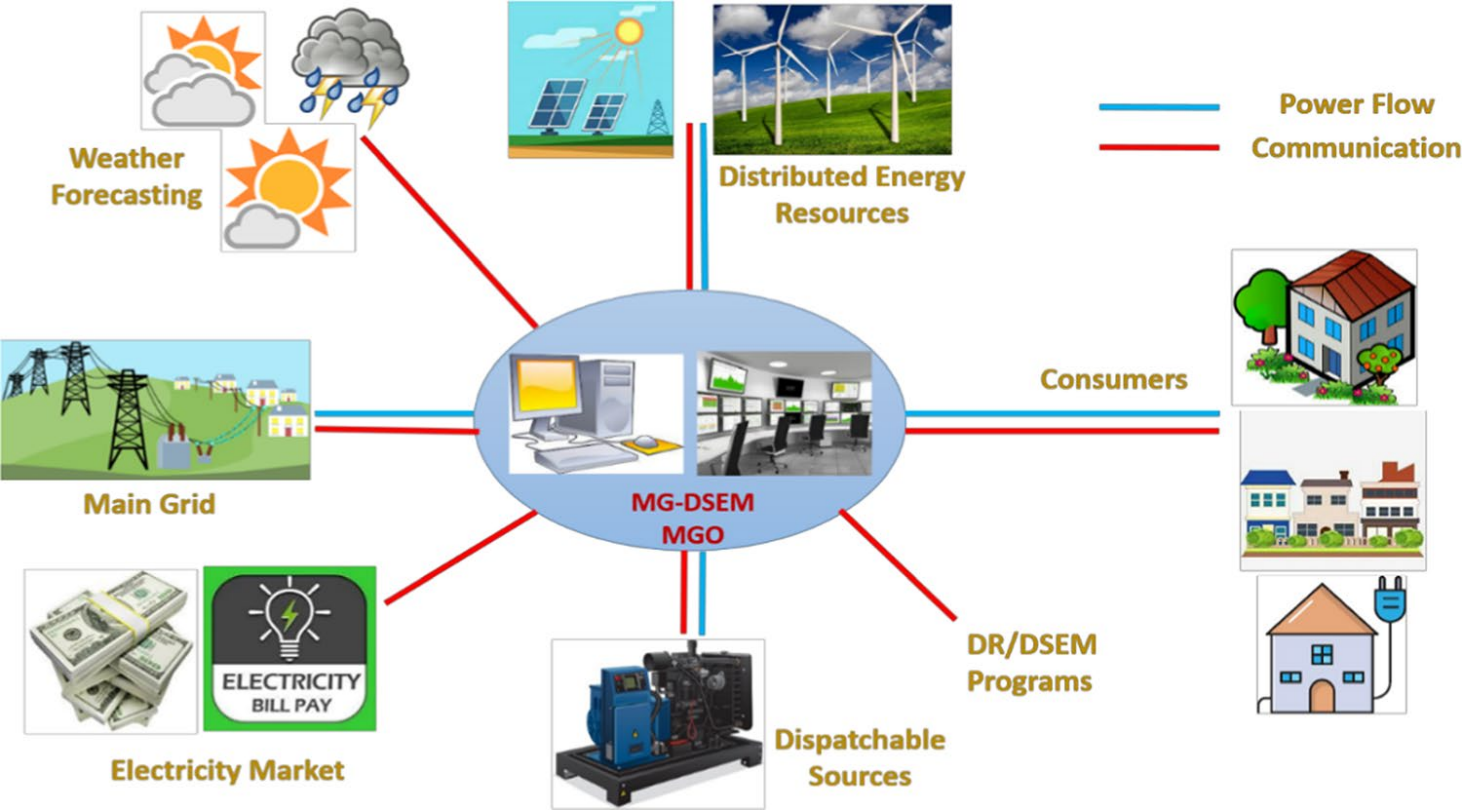
Distribution network operator (DNO) system.

Distribution network operator (DNO) is responsible for effectively managing and controlling distribution network operation achieving an optimal power sharing between different distributed generation resources and the power received from the grid. Additionally, the DNO and consumers collaborate using demand response programs to determine the optimal energy not supplied, and the consumer’s optimal incentive rate as elaborated in Fig. 3.

**Fig. 4:**
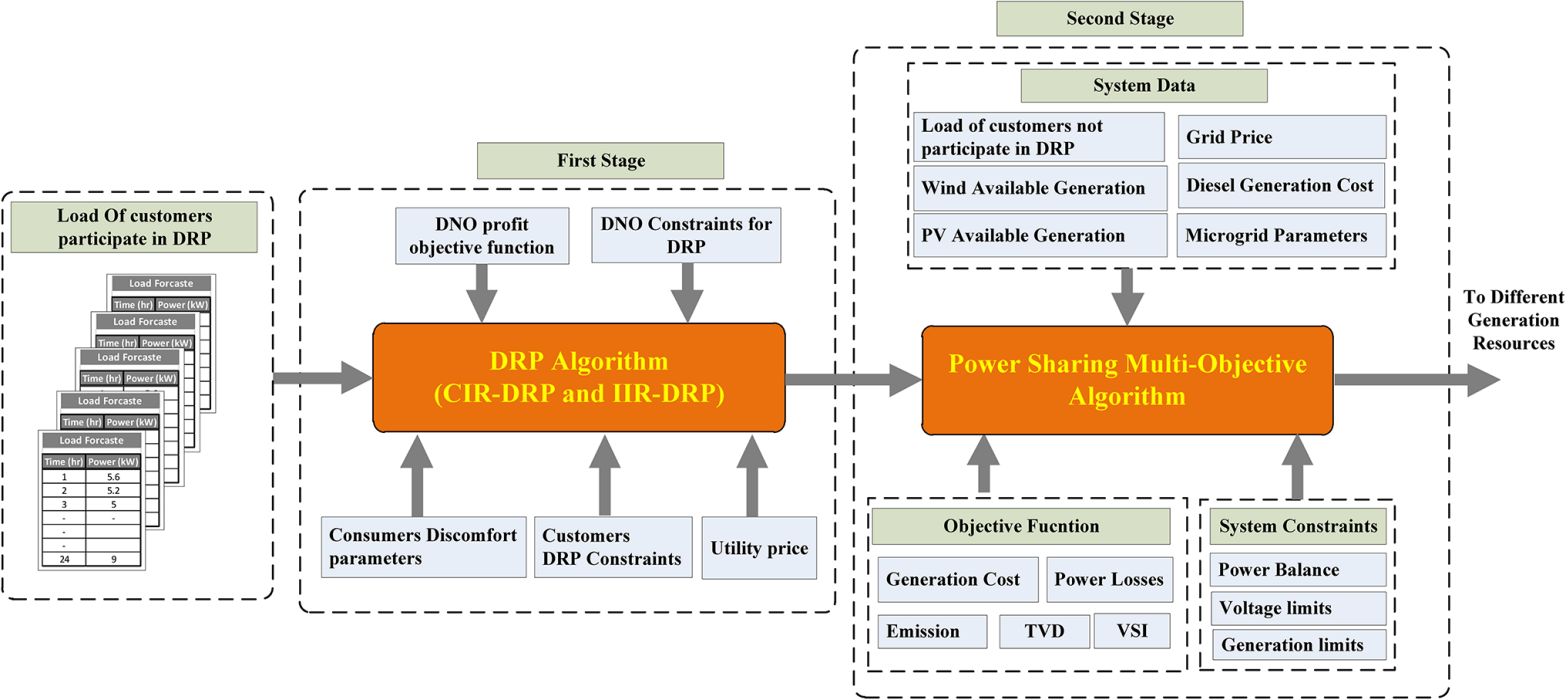
Proposed operation framework.

The proposed framework involves a two-stage optimization approach as illustrated in Fig. 4. The first stage of the proposed strategy is applying Elephant Herding Optimization (EHO) technique using MATLAB software as a mathematical model to maximize the DNO profit by optimizing the consumer incentive rate and curtailed power while considering the constraints associated with the DRP. Also, in the second stage of the proposed strategy, EHO combined with the technique for order of preference by similarity to ideal solution (TOPSIS) approach is constructed and applied on MATLAB software for multi-objective optimal power sharing between DGRs and the grid to minimize the total energy losses, improving the voltage profile and maximizing the voltage stability index. Additionally, reducing the gas emissions and minimizing the total operational cost of the distribution network considering the operational constraints. To ensure the effectiveness of the proposed framework, IEEE 33 bus radial distribution network has been used and the load flow calculations using MATLAB software have been applied using the backward/forward method which is more suitable for radial distribution system.

## Modelling of the operational framework

This section presents modelling of the proposed framework. Firstly, this section presents the modelling of demand response program considering DRP objective function and its constraints. Secondly, the modelling of multi-objective optimal power sharing between DGRs and the grid considering the operational constraints.

### First stage: demand response program modelling

Demand Response programs (DRP) promote the reduction of energy consumption among energy consumers to ensure the reliable functioning of micro-grids. DRP offer consumers the opportunity to actively contribute to the functioning of the electric grid by adjusting their electricity usage during peak periods. By participating in these programs, consumers receive financial incentives. The optimal DRP determines the incentive rate for consumers to maximize the profit of the DNO, while satisfying the DRP’s constraints. However, some of consumer’s discomfortability may be caused when there is a decreasing in energy demand. Consumer’s discomfortability ($\:{{{\uppsi\:}}_{\text{j}}}^{\text{h}}$) exponentially increased when the consumer’s curtailed power increased, as depicted in Eq. (1). Where: ($\:{{\beta}}_{\text{j}}$) represents the consumers’ ability to cope with uncomfortable situations. Consequently, consumers who have the lower ability about withstanding the curtailed power will have a higher value of ($\:{{\beta}}_{\text{j}}$), $\:{{\text{P}\text{N}\text{S}}_{\text{j}}}^{\text{h}}$ denotes the power curtailed by consumer j during the $\:{\text{h}}^{\text{t}\text{h}}$interval (kW), relative to the total actual power demand of consumer j ($\:{{\text{P}}_{\text{j}}}^{\text{h}})$.

1 $$\:{{{\uppsi\:}}_{\text{j}}}^{\text{h}}={e}^{{{\upbeta\:}}_{\text{j}}\left(\frac{{{\text{P}\text{N}\text{S}}_{\text{j}}}^{\text{h}}}{{{\text{P}}_{\text{j}}}^{\text{h}}}\right)}-1$$

Consumer participation in DRP can lead to increased profits for DNO. DNO’s role in DRP involves determining the optimal incentive rates ($$\:{\gamma\:}^{h}$$) for participating consumers, given ($$\:\text{a}$$). The profit for DNO can be calculated using Eq. (2), where: $$\:{\text{a}}^{\text{h}}$$ represents the energy price of power grid ($/MWh). $$\:\left[a\:{{\text{P}\text{N}\text{S}}_{\text{j}}}^{\text{h}}\right]$$ Represents the money saved by power not supplied from the grid, while [$$\:{{\upgamma\:}}^{\text{h}}{{\text{P}\text{N}\text{S}}_{\text{j}}}^{\text{h}}]$$ represents the total consumer’s incentive about their curtailed power.

2 $$\:C={\sum\:}_{\text{j}=1}^{\text{J}}\left(\left[a\:{{\text{P}\text{N}\text{S}}_{\text{j}}}^{\text{h}}\right]-\left[{{\upgamma\:}}^{\text{h}}{{\text{P}\text{N}\text{S}}_{\text{j}}}^{\text{h}}\right]\right)$$

The consumer ‘s involvement in the demand response program is determined by the incentive received and the level of discomfort experienced, which is defined as the consumer’s benefit in Eq. (3). Therefore, the Consumers will participate in DRP only when their benefits are positive. Also, Table 2 provides information about the locations of the participating consumers, their demand load, and their inability factor ($\:{{\beta}}_{\text{j}}$ (β_j_) to handle uncomfortable situations.

3 $$\:{\text{C}\text{B}}_{\text{j}}=\left[{\gamma\:}^{h}{{\text{P}\text{N}\text{S}}_{\text{j}}}^{\text{h}}\right]-{{\psi\:}_{j}}^{h}$$

**Table 2:** Details for Number of Five consumers participate in DRP^27^.

	Consumer Location	Demand Load (kW)	β
$\:{C}_{1}$	Bus 9	60	1
$\:{C}_{2}$	Bus 14	120	2
$\:{C}_{3}$	Bus 22	90	2
$\:{C}_{4}$	Bus 25	420	3
$\:{C}_{5}$	Bus 30	200	3

#### Demand response program objective function

The maximization of DNO profit can be achieved by determining the optimal Power Not Supplied (PNS) for each consumer participating in DRP, as well as the Optimal Incentive Rate. The DNO can apply the same incentive rate for all consumers or a different incentive rate for each consumer. Equation (4) demonstrates that the maximization of DNO profit using Fixed Incentive Rate for all consumers. Where ($\:{{\upgamma\:}}^{\text{h}}$) is an incentive rate for all consumers participate in DRP at ( $\:{h}^{th}$) interval, $\:{{\text{P}\text{N}\text{S}}_{\text{j}}}^{\text{h}}$, is the power not supplied of j^th^ consumer at $\:{h}^{th}$ interval, and (a) is the electricity market price.

4 $$\:{DRP}_{1}=\sum\:_{\text{h}=1}^{\text{H}}{\sum\:}_{\text{j}=1}^{\text{J}}\left(\:\left[a\:{{{*}\:\text{P}\text{N}\text{S}}_{\text{j}}}^{\text{h}}\right]-\left[{{\upgamma\:}}^{\text{h}}*\:{{\text{P}\text{N}\text{S}}_{\text{j}}}^{\text{h}}\right]\right)$$

Equation (5) demonstrates that the maximization of DNO profit can be achieved by identifying the optimal Power Not Supplied (PNS) using a different Incentive rate for each consumer. Where ($\:{{{\upgamma\:}}_{\text{j}}}^{\text{h}}$) is an incentive rate for j^th^ consumer participate in DRP at ($\:{h}^{th}$) interval.

5 $$\:{DRP}_{2}=\sum\:_{\text{h}=1}^{\text{H}}{\sum\:}_{\text{j}=1}^{\text{J}}\left(\:\left[\:a\text{*}\:{{\text{P}\text{N}\text{S}}_{\text{j}}}^{\text{h}}\right]-{[{{\upgamma\:}}_{\text{j}}}^{\text{h}}*{{\text{P}\text{N}\text{S}}_{\text{j}}}^{\text{h}}]\right)$$

#### Demand response program constraints

To attain an optimal Power Not Supplied (PNS) for every consumer and an optimal incentive rate, the subsequent limitations must be considered.

I. Power curtailment limit.

To avoid complete shutdown of consumer demand power at any instant, the power not supplied of each consumer at interval h shall be limited as given in Eq. (6). Where: $\:{\text{P}\text{N}\text{S}}_{j}^{min,h}\:and\:\:{\text{P}\text{N}\text{S}}_{j}^{max,h}$ represent the permissible maximum and minimum power not supplied, which are percentage of the actual load power as given by Eqs. (7 and 8) respectively. In this study,$\:\:{\mu\:}_{1}$ and $\:{\mu\:}_{2}$ are assigned values of 0 and 0.4 respectively^40^.

6 $$\:{\text{P}\text{N}\text{S}}_{j}^{min,h}\le\:{\text{P}\text{N}\text{S}}_{j}^{h}\le\:{\text{P}\text{N}\text{S}}_{j}^{max,h}\:\:\:\:\:\:\:\:\:\:h={1,2},3,\ldots H$$

7 $$\:{\text{P}\text{N}\text{S}}_{\text{j}}^{\text{m}\text{i}\text{n},\text{h}}\:=\:\:{{\upmu\:}}_{1}\:\:\:{\text{P}}_{\text{j}}^{\text{h}}$$

8 $$\:{\text{P}\text{N}\text{S}}_{\text{j}}^{\text{m}\text{a}\text{x},\text{h}}\:=\:\:\:{{\upmu\:}}_{2}\:\:{\text{P}}_{\text{j}}^{\text{h}}$$

II. Incentive rate limit.

The DNO ‘s incentive rate is designed to guarantee a profitable outcome for the DNO. To achieve this, it is crucial to maintain the incentive rate within the specified as given by Eq. (9), where $\:{\gamma\:}^{max}\:and\:{\gamma\:}^{min}$ represent the maximum and minimum incentive rates, respectively. The upper and lower boundaries of this range are determined by the utility energy price (a), as indicated in Eqs. (10 and 11), where: $\:{\upeta\:}$ is factory between 0 and 1.

9 $$\:{\gamma\:}^{min}\le\:\:{\gamma\:}^{h}\:\le\:{\gamma\:}^{max}\:\:\:\:\:\:\:\:h={1,2},\ldots,H$$

10 $$\:{\gamma\:}^{max}=\text{m}\text{i}\text{n}\left(a\right)$$

11 $$\:{\gamma\:}^{min}={\upeta\:}\:\text{m}\text{i}\text{n}\left(a\right)$$

III. Individual consumer benefit.

Every consumer will receive a certain incentive to overcome the discomfort associated with curtailed power Therefore, the Consumers will participate in DRP only when their benefits is a positive value as given by Eq. (12).

12 $$\:(\left[{\gamma\:}^{h}\:{\text{P}\text{N}\text{S}}_{j}^{h}\right]-[{\psi\:}_{j}^{h}\left]\:\right)>\:0$$

IV. DNO budget limit.

The total incentive provided to all consumers along the day shall be within the schedule budget of the DNO as defined by Eq. (13). Where: $\:\text{D}\text{N}\text{B}$ is the daily DNO budget.

13 $$\:\sum\:_{\text{j}=1}^{\text{J}}\sum\:_{\text{h}=1}^{\text{H}}{[{\upgamma\:}}_{\text{j}}^{\text{h}}\:{\text{P}\text{N}\text{S}}_{\text{j}}^{\text{h}}]\:\le\:\text{D}\text{N}\text{B}\:$$

### Second stage: multi-objectives optimal power sharing

This segment represents the optimal distributed generation resources operation (ODGRO) problem that incorporates various DGR technologies with the goal of optimizing multiple significant objectives.

#### Optimal power sharing operational objectives

In practical terms, distribution companies are required to accomplish multiple objectives. As a result, this power dispatching process considers five objectives for the distribution generators (DGs) and the utility grid.

I. Power losses minimization.

The distribution systems have traditionally been the main source of power loss during power delivery. As a result, utilities prioritize minimizing power losses as shown in Eq. (14). Where: $\:{\text{P}}_{\text{i}}\:,\:{\text{P}}_{\text{j}}\:,\:{\text{Q}}_{\text{i}}\:\text{a}\text{n}\text{d}\:{\text{Q}}_{\text{j}}$ are Active and reactive power at buses $\:{\text{i}}_{\text{t}\text{h}}$ and $\:{\text{j}}_{\text{t}\text{h}}$ respectively, N is the total number of Buses and $\:{V}_{i}<{\delta\:}_{i}$ and $\:{V}_{j}<{\delta\:}_{j}$ is bus i, and bus j voltage respectively. $\:{r}_{ij}\:,\:\:{x}_{ij}\:$ are element of $\:{ij}_{th}\:$line resistance and reactance respectively.

14 $$\:{\text{f}}_{1}\:=\text{M}\text{i}\text{n}\:\left[\sum\:_{\text{i}=1}^{\text{N}}\sum\:_{\text{j}=1}^{\text{N}}{{\upalpha\:}}_{\text{i}\text{j}}\:\left({\text{P}}_{\text{i}}\:{\text{P}}_{\text{j}}+{\text{Q}}_{\text{i}}\:{\text{Q}}_{\text{j}}\right)+\:{{\upbeta\:}}_{\text{i}\text{j}}\:\left({\text{Q}}_{\text{i}}\:{\text{P}}_{\text{j}}-\:{\text{P}}_{\text{i}}\:{\text{Q}}_{\text{j}}\right)\right]$$

15 $$\:{{\upalpha\:}}_{\text{i}\text{j}}=\:\frac{{\text{r}}_{\text{i}\text{j}}}{{\text{V}}_{\text{i}}\:{\text{V}}_{\text{j}}}\:\text{cos}\:\left(\:{{\updelta\:}}_{\text{i}}-\:{{\updelta\:}}_{\text{j}}\:\right)\:\:$$

16 $$\:{{\upbeta\:}}_{\text{i}\text{j}}=\:\frac{{\text{r}}_{\text{i}\text{j}}}{{\text{V}}_{\text{i}}\:{\text{V}}_{\text{j}}}\:\text{sin}\:(\:{{\updelta\:}}_{\text{i}}-\:{{\updelta\:}}_{\text{j}}\:)$$

II. Voltage deviation minimization.

The quality of supply voltage is becoming a growing concern for the modern power system. Hence, the proposed framework optimizes the voltage deviation along the distribution system as given by Eq. (17)

17 $$\:{\text{f}}_{2}=\:\text{M}\text{i}\text{n}\:\left[\sum\:_{\text{i}=1}^{\text{N}}{\left(\:{\text{V}}_{\text{i}}-1\right)}^{2}\right]\:\:$$

III. Voltage stability index (VSI) maximization.

VSI represents the power network security which shows how well a node can keep its voltage profile within allowable bounds under various high loading scenarios^46^. The VSI of the line connected between bus i and bus j is given by Eq. (18).

18 $$\:{\text{V}\text{S}\text{I}}_{\text{i}\text{j}}={{\text{V}}_{\text{j}}}^{4}-4\:\left({\text{P}}_{\text{i}}\:{\text{r}}_{\text{i}\text{j}}+\:{\text{Q}}_{\text{i}}{\text{x}}_{\text{i}\text{j}}\right)\:{{\text{V}}_{\text{j}}}^{2}-4\:{\left(\:{\text{P}}_{\text{i}}\:{\text{x}}_{\text{i}\text{j}}-\:{\text{Q}}_{\text{i}}\:{\text{r}}_{\text{i}\text{j}}\:\right)}^{2}$$

The proposed framework maximizes the VSI of a line with the minimum VSI value as shown in Eq. (19). to improve stability margin of the network voltage

19 $$\:{\text{f}}_{3}=\text{Max\:}[\text{min}\:\left(\:{\text{V}\text{S}\text{I}\:}_{\text{i}\text{j}}\right)]$$

IV. Generation and operational cost minimization.

The proposed framework optimizes the total generation cost as given by Eq. (20). Where, the first term is the price of power received from grid and the second term is the generation price of DGRs within the network. Where$\:\:{{P}_{Grid}}^{h}$ is the power received from the grid at hour h. $\:{\text{P}}_{{\text{G}}_{\text{j}}}\:\text{i}\text{s}\:$ active power of the distributed generator j. $\:{\text{a}}_{\text{j}}$,$\:{\text{b}}_{\text{j}}$, $\:{\text{c}}_{\text{j}}$ are cost function coefficients for $\:{DG}_{j}$. The cost function of DGRs’s coefficients are illustrated in Table 3.

20 $$\:{\:f}_{4}=Min\:\left[\right(a{\:}{{P}_{Grid}}^{h}+{\sum\:}_{j\varepsilon\:{N}_{G}}{a}_{j}{{\:P}^{2}}_{G}{+}{b}_{j}{\:P}_{G}+{C}_{j}\text{)]}$$

**Table 3:** Data of distributed Energy resources connected to IEEE 33-BUS DS in^13^.

Energy resources	$$\:{a}_{j}$$	$$\:{b}_{j}$$	$$\:{C}_{j}$$
	$/M$$\:{W}^{2}h$$	$/MWh	$/h
PV 1	-	2.3	-
PV 2	-	2.3	-
Diesel Gen 1	20	100	12
Wind Turbine	-	1.9	-
Diesel Gen 2	20	100	12

##  Gasses emission minimization

Diesel generators emit a large amount of gasses which represent a harmful effect on the peoples and animals and the life. The most dangerous types of this gasses are $$\:{CO}_{2}$$, $$\:{NO}_{X}$$ and $$\:{SO}_{x}$$. So, the government encourage the DNO which have a number of DGRs to minimize the gas emissions versus an incentive rate in order to control the amount of gas emissions by the energy management optimization techniques which minimize the power sharing by diesel generators to limit the gas emissions. The proposed framework optimizes the emission gases of the bio-mass based distributed generators as given by Eq. (23). Where: $$\:K$$ is number of pollution gases from Diesel Generator $$\:\left(\:{DG}_{m}\:\right)\:$$ [$$\:{\:CO\text{\:}}_{2}$$,$$\:{\:SO\text{\:}}_{2}\:$$and$$\:{\:NO}_{2}$$], $$\:{\text{R}}_{\text{K}\:}$$is the weighting factor of pollution gas $$\:K$$ ($/kg), $$\:{\text{K}}_{\text{E}\text{N}\text{V}}$$ is the amount of gas $$\:K$$ emission of DG (kg/kWh) and $$\:{\:\text{P}}_{\text{M}}$$ is the Output power of$$\:{\:DG}_{m}$$. The amount of gas emission from DG and weighting factor are given in Table 4.

21 $$\:{f}_{5}=Min\:[\sum\:_{m=1}^{M}\sum\:_{k=1}^{K}{\text{R}}_{\text{K}\:\:\:}{\text{K}}_{\text{E}\text{N}\text{V}\:}\text{\:}{\text{P}}_{\text{M}\:}\:]$$

**Table 4:** Emission parameters of distributed generations in^15,18^.

K	Weighting factor ($\:{\text{R}}^{\circ}_{\text{K}})$	Emission Factor (Kg/KWh)		
		PV	WT	DG
$CO^{\circ}_{2}$	0.021	0	0	0.6444
$SO^{\circ}_{2}$	1.4842	0	0	0.000204
$NO^{\circ}_{2}$	6.2964	0	0	0.00981

### Optimal power sharing operational constraints

The optimal operation of the network while adhering to technical constraints, such as those relating to voltage and power balance, generation boundaries, and other constraints related to the demand response program.

I. Power balance equation.

The combined power generated by DGRs and the power received from the grid shall equal the total demand load of the network including the power losses, as depicted in Eq. (24). Where, $\:\:{\text{P}}_{\text{G}\text{r}\text{i}\text{d}}^{\text{h}}\:$represents the power received from the grid during the $\:{h}^{th}$ interval, $\:{\text{P}}_{\text{S}\text{o}\text{l}\text{a}\text{r}}^{\text{h}},\:{\text{P}}_{\text{D}\text{G}}^{\text{h}},{\text{P}}_{\text{W}\text{i}\text{n}\text{d}}^{\text{h}}\:$denote the photo voltaic system output power, conventional DG, wind sources, and power losses respectively, during the $\:{h}^{th}$ interval. It shall be noted, the load curtailment ($\:{\text{P}\text{N}\text{S}}_{\text{j}}^{\text{h}}$) due to DRP program is deducted from the original load ($\:{\text{P}}_{\text{j}}^{\text{h}}$) as shown in Eq. (22).

22 $$\:{\text{P}}_{\text{G}\text{r}\text{i}\text{d}}^{\text{h}}+\sum\:_{\text{j}=1}^{{\text{N}}_{\text{s}}}{\text{P}}_{\text{S}\text{o}\text{l}\text{a}\text{r}}^{\text{h}}+\sum\:_{\text{j}=1}^{{\text{N}}_{\text{D}\text{G}}}{\text{P}}_{\text{D}\text{G}}^{\text{h}}+\sum\:_{\text{j}=1}^{{\text{N}}_{\text{W}}}{\text{P}}_{\text{W}\text{i}\text{n}\text{d}}^{\text{h}}\:=\sum\:_{\text{j}=1}^{{\text{N}}_{\text{c}}}{\text{P}}_{\text{j}}^{\text{h}}-\sum\:_{\text{j}=1}^{\text{J}}{\text{P}\text{N}\text{S}}_{\text{j}}^{\text{h}}+{\text{P}}_{\text{L}\text{o}\text{s}\text{s}}^{\text{h}}\:\:\:\:\:\:\:\:\:\:\:\text{h}=\text{1,2},3,\dots\:,\text{H}$$

II. Generation limit.

The generation resources shall operate within the permissible safe operation boundaries as given by Eq. (23). Where: $\:{P}_{GR}^{max},\:{P}_{GR}^{min}$ are the maximum and the minimum allowable generation power of generation units.

23 $$\:{\text{P}}_{\text{G}\text{R}}^{\text{m}\text{i}\text{n}}\le\:{\text{P}}_{\text{G}\text{R}}^{\text{h}}\le\:{\text{P}}_{\text{G}\text{R}}^{\text{m}\text{a}\text{x}}\:\:\:\:\:\:\:\:\:\:\text{h}=\text{1,2},3,\ldots,\:\text{H}$$

III. Voltage constraints.

The voltage level at every node must be limited within the permissible range [95–105%] of the system nominal voltage to ensure the secure functioning of both the load and generation, as given by Eq. (24).

24 $$\:{\text{V}}^{\text{m}\text{i}\text{n}}\le\:{\text{V}}_{\text{i}}^{\text{h}}\le\:{\text{V}}^{\text{m}\text{a}\text{x}}\:\:\:\:\:\:\:\:\:\:\text{h}=\text{1,2},3,\ldots,\:\text{H}$$

## Proposed strategy

The proposed framework applies the Technique for Order of Preference by Similarity to Ideal Solution (TOPSIS) and elephant Herding Optimization (EHO) to achieve an efficient and effective operation of distributed energy resources. TOPSIS determines the optimal solution depend on the preferences and similarities to an ideal solution. EHO aids in finding an optimal solution of the TOPSIS by mimicking the behavior of elephant herds^40^.

### Multi-objective formulation using TOPSIS approach

The TOPSIS approach is applied for multi-objective problem of distribution systems as shown in objectives mentioned in Eqs. (14, 17, 19, 20, and 21) as shown in Eq. (25). Each objective is called attribute of the solution.

25 $$\:\:\text{O}\text{p}\text{t}\text{i}\text{m}\text{i}\text{z}\text{e}\:\left[{f}_{1}\left(x\right),{f}_{2}\left(x\right),\dots\:\dots\:\dots\:.{f}_{{n}_{2}}\left(x\right)\right]$$

The TOPSIS methodology relies on the principles of Euclidean geometry, wherein two base points are employed to identify the optimal trade-off solution referred to as the Positive Ideal Solution (PIS) and Negative Ideal Solution (NIS). Consequently, the chosen various solution should have boundary limits of PIS and NIS. This approach enables the solutions should be focused on their respective best solutions. The fundamental steps involved in the TOPSIS are outlined as follows:

1. For $\:m$ solutions determine the objective function $\:\text{n}\:$and arrange the solution in matrix form as given by Eq. (28) where: $\:{f}_{ij}$ is the value of the $\:{i}_{th}$ alternate of the $\:{j}_{th}$ objective.
2. A normalized matrix is formulated to normalize the solution’s attributes using Eq. (27)
  26 $$\:\text{F}=\left[\begin{array}{cccc}{\text{f}}_{11}&\:{\text{f}}_{\text{i}2}&\:\dots\:\dots\:\dots\:&\:{\text{f}}_{1\text{n}}\\\:{\text{f}}_{21}&\:{\text{f}}_{22}&\:\dots\:\dots\:\dots\:&\:{\text{f}}_{2\text{n}}\\\:.&\:.&\:.&\:.\\\:.&\:.&\:.&\:.\\\:{\text{f}}_{\text{m}1}&\:{\text{f}}_{\text{m}2}&\:\dots\:\dots\:\dots\:&\:{\text{f}}_{\text{m}\text{n}}\end{array}\right]$$
  27 $$\:{\text{r}}_{\text{i}\text{j}}=\:\frac{{\text{f}}_{\text{i}\text{j}}}{\sqrt{{\sum\:}_{\text{i}=1}^{\text{m}}{{\text{f}}_{\text{i}\text{j}}}^{2}}}$$
3. To introduce different weights for the objective function, a weighted organized making choices matrix is formulated as illustrated in Eq. (28). Where: $\:{w}_{j}$ is the weight of the $\:{j}_{th}$ objective and $\:\:\sum\:_{j=1}^{{n}_{2}}{w}_{j}$ = 1.
  28 $$\:{\text{v}}_{\text{i}\text{j}}=\:{\text{w}}_{\text{j}}\:{\text{r}}_{\text{i}\text{j}}$$
4. Determine the Positive Ideal Solution (PIS) and Negative Ideal Solution) NIS (corresponding to the best and worst results of each single objective, respectively, as given by Eqs. (29 and 30).
  29 $$\:PIS=\left\{{{v}_{1}}^{+},{{v}_{2}}^{+},{{v}_{3}}^{+},\dots\:\dots\:\dots\:\dots\:.,{{v}_{n}}^{+}\right\}$$
  30 $$\:NIS=\left\{{{v}_{1}}^{-},{{v}_{2}}^{-},{{v}_{3}}^{-},\dots\:\dots\:\dots\:\dots\:.,{{v}_{n}}^{-}\right\}$$
  The individuals of PIS and NIS are selected according to Eqs. (31 and 32) respectively.
  31 $$\:{{\text{v}}_{\text{j}}}^{+}=\left\{\begin{array}{cc}\text{m}\text{a}\text{x}\left({\text{v}}_{\text{i}\text{j}}\right)&\:\text{f}\text{o}\text{r}\:\text{o}\text{b}\text{j}\text{e}\text{c}\text{t}\text{i}\text{v}\text{e}\:\text{j}\:\text{m}\text{a}\text{x}\text{i}\text{m}\text{i}\text{z}\text{a}\text{t}\text{i}\text{o}\text{n}\\\:\text{m}\text{i}\text{n}\left({\text{v}}_{\text{i}\text{j}}\right)&\:\text{f}\text{o}\text{r}\:\text{o}\text{b}\text{j}\text{e}\text{c}\text{t}\text{i}\text{v}\text{e}\:\text{j}\:\text{m}\text{i}\text{n}\text{i}\text{m}\text{i}\text{z}\text{a}\text{t}\text{i}\text{o}\text{n}\end{array}\:\:\:\right.$$
  32 $$\:{\:{\text{v}}_{\text{j}}}^{-}=\left\{\begin{array}{cc}\text{m}\text{a}\text{x}\left({\text{v}}_{\text{i}\text{j}}\right)&\:\text{f}\text{o}\text{r}\:\text{o}\text{b}\text{j}\text{e}\text{c}\text{t}\text{i}\text{v}\text{e}\:\text{j}\:\text{m}\text{i}\text{n}\text{i}\text{m}\text{i}\text{z}\text{a}\text{t}\text{i}\text{o}\text{n}\\\:\text{m}\text{i}\text{n}\left({\text{v}}_{\text{i}\text{j}}\right)&\:\text{f}\text{o}\text{r}\:\text{o}\text{b}\text{j}\text{e}\text{c}\text{t}\text{i}\text{v}\text{e}\:\text{j}\:\text{m}\text{a}\text{x}\text{i}\text{m}\text{i}\text{z}\text{a}\text{t}\text{i}\text{o}\text{n}\end{array}\:\:\:\right.$$
5. For each solution, The Euclidean distances $\:{d}_{i+}\:$and $\:{d}_{i-}\:$from the positive and best solutions using Eqs. (33 and 34) respectively.
  33 $$\:{{\text{d}}_{\text{i}}}^{+}=\:\sqrt{{\sum\:}_{\text{j}=1}^{\text{n}}{\left({\text{v}}_{\text{i}\text{j}}-\:{{\text{v}}_{\text{j}}}^{+}\right)}^{2}\:}\:$$
  34 $$\:{{\text{d}}_{\text{i}}}^{-}=\:\sqrt{{\sum\:}_{\text{j}=1}^{\text{n}}{\left({\text{v}}_{\text{i}\text{j}}-\:{{\text{v}}_{\text{j}}}^{-}\right)}^{2}}$$
6. The relative closeness index (RCI) for each solution is determined as shown in Eq. (35). The best solution is the highest RCI.
  35 $$\:{{\text{C}}_{\text{i}}}^{+}=\:\frac{{{\text{d}}_{\text{i}}}^{+}}{{{\text{d}}_{\text{i}}}^{+}+\:{{\text{d}}_{\text{i}}}^{-}}$$

### Elephant herd optimization (EHO)

**Fig. 5:**
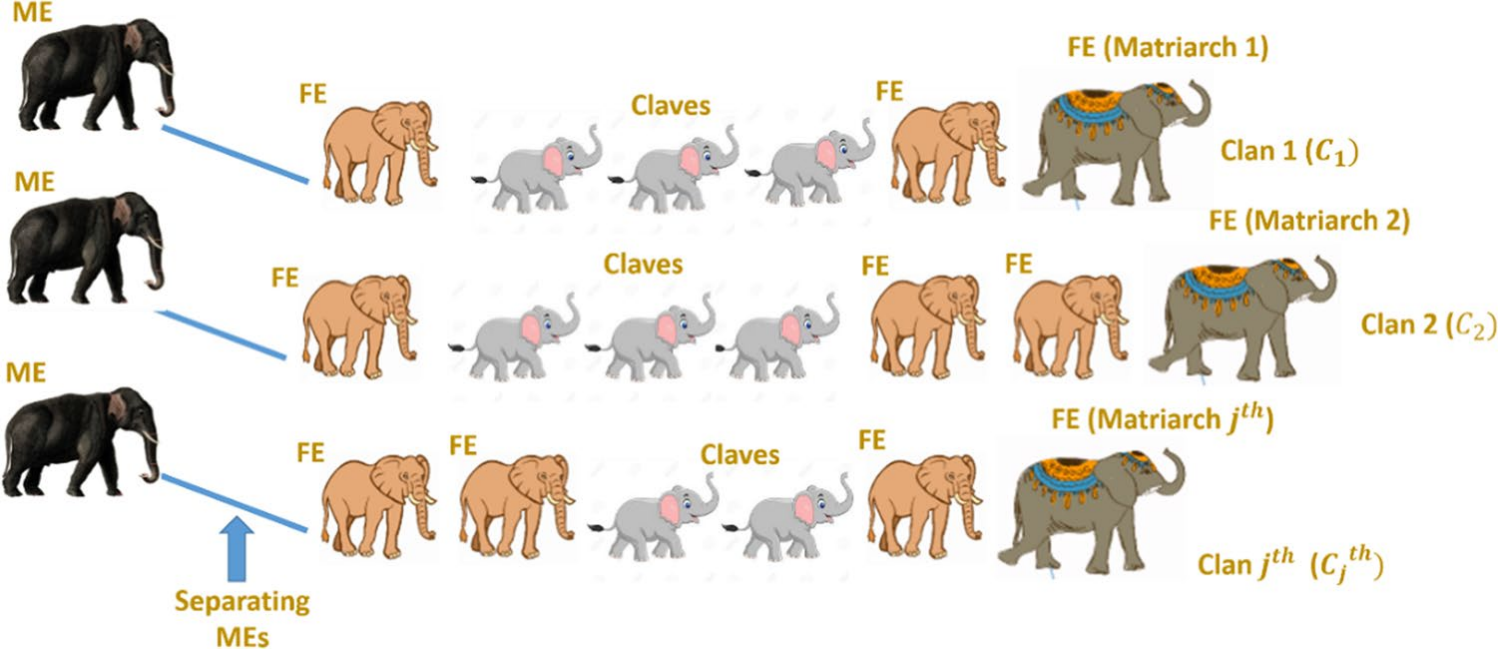
Environmental behavior of elephants simulated in EHO.

Elephant herding behavior is emulated via the EHO algorithm, which is an optimization technique based on environmental inspiration. Specifically, the female elephants (FEs) protect their young from predatory animals. That FEs communicate through seismic signals produced by foot walking. These waves travel through the ground and can be sensed by elephants, serving as warning signals for potential danger. Elephants are known to be social animals, forming herds consisting of multiple groups of FEs and their young, as depicted in Fig. 5. Within each herd, a matriarch or leader elephant influences the movement of the clans. FEs typically stay with their groups, while male elephants (MEs) separate from their families as they mature and maintain contact through low-frequency vibrations. It is assumed that each group or clan contains the same number of members or elephants. The matriarch’s group determine the best solution within the group, while the position of the MEs’ group represents the worst solution^46^. EHO technique is mathematically modeled in the following steps.

**Step 1 (Elephant’s position update)**.

Every elephant in the clan, with the exception of the matriarch and ME, is updated with their relative positions for the best and worst options as given by Eq. (38). Where: $\:{Z}_{cj,i\:\:}$and $\:{Z}_{new,cj,i\:\:}$are the old and the new positions of the $\:\:{i}_{th\:}$ elephant in the $\:{C}_{j,th\:}\:$clan respectively.$\alpha$α is the scaling factor situated between 0 and 1. Whereas, the best position of the $\:\:{i}_{th\:\:}$elephant in the $\:{C}_{j,th\:}$clan is $\:{Z}_{best,cj,i\:\:}$and r is a random number between [0 1].

36 $$\:{Z}_{new,cj,i}=\:{Z}_{cj,i}+\:\alpha\:\:\left(\:{Z}_{best,cj,i}-\:{Z}_{cj,i}\:\right)\:r\:$$

**Step 2 (Matriarch’s Position update)**.

The location of the best solution represents the matriarch of the clan. This position is updated using the center of the clan $\:\left(\:{Z}_{center,cj}\right)$ as given by Eq. (37). Where, $\:\rho\:$ is the scale factor in the interval [0,1]. The center of the clan is calculated from the position of all individuals as given by Eq. (38). Where$\:\:{n}_{z\:\:}$is the number of elephants in each group.

37 $$\:{\text{Z}}_{\text{n}\text{e}\text{w},\text{c}\text{j},\text{i}}=\:{\uprho\:}\:{\text{Z}}_{\text{c}\text{e}\text{n}\text{t}\text{e}\text{r},\text{c}\text{j}}$$

38 $$\:{\text{Z}}_{\text{c}\text{e}\text{n}\text{t}\text{e}\text{r},\text{c}\text{j}}=\sum\:_{\text{i}=1}^{\text{n}}{\text{Z}}_{\text{c}\text{j},\text{i}}/{\text{n}}_{\text{z}}$$

Updating the matriarch position using center of the clean may cause solution divergence away from the global solution. Therefore, in EHO the location of matriarch elephants is updated to be in the vicinity of the current best position as shown in Eq. (39). Where: $\:{Z}_{best,cj,i\:\:}$is the current best location determined by the matriarch elephants of each group.

39 $$\:{\text{Z}}_{\text{n}\text{e}\text{w}\:,\:\text{c}\text{j},\text{i}}=\:{\text{Z}}_{\text{b}\text{e}\text{s}\text{t},\text{c}\text{j},\text{i}}+\:{\uprho\:}\:{\text{Z}}_{\text{c}\text{e}\text{n}\text{t}\text{e}\text{r},\text{c}\text{j}}$$

**Step 3 (Males separation)**.

The worst elephants are separated from their family groups. To attain the same number of elephants in the clan, the separated males are compensated with new elephant babies. The position of these babies is given by Eq. (40). Where: $\:{Z}_{worst,cj,i\:\:}$is the new babies’ position in the $\:{c}_{jth\:\:}$clan. $\:{Z}_{max\:\:},\:{Z}_{min\:\:}$ are the maximum and minimum boundaries for each clan or group.

40 $$\:{Z}_{worst,\:cj,i}=\:{Z}_{min}+r\left({Z}_{max}-{Z}_{min}+1\right)$$

According to Eq. (39), the newly babies of elephants will take a randomly selected location. However, it is noted that the elephants let their young near to the stronger females to keep them from predatory animals. Hence, the newly generated elephants are assumed to be in a location near to a stronger female as given by Eqs. (41 and 42). Where: µ is random proximity factor between [0.9–1.1] and $\:{Z}_{local,cj}$ is the local best position of the elephant of the $\:{{c}_{j}}_{th}$ clan.

41 $$\:{\text{Z}}_{\text{w}\text{o}\text{r}\text{s}\text{t},\text{c}\text{j},\text{i}\:\:}=\:\:{\:\:\text{Z}}_{{\text{f}\text{i}\text{t}\text{n}\text{e}\text{s}\text{s}}_{\text{c}\text{j}}}$$

42 $$\:{\:\text{Z}}_{{\text{f}\text{i}\text{t}\text{n}\text{e}\text{s}\text{s}}_{\text{c}\text{j}}}=\:{\upmu\:}\:\:{\text{Z}}_{\text{l}\text{o}\text{c}\text{a}\text{l},\text{c}\text{j}}\:$$

**Step 4 (Convergence and stopping)**.

Repeat step 1 to step 4 till the convergence or stopping conditions is satisfied.

### TOPSIS and EHO multi-objective technique

As discussed in the previous sections, the optimal demand response program and optimal power sharing problems are nonlinear multiple objectives. This complexity leads to the generation of numerous solutions, making it necessary to employ an efficient approach to identify the most suitable solution from the pool of competitive options. On the other hand, EHO is a single objective algorithm. Consequently, the TOPSIS approach is employed in conjunction with the EHO to facilitate the selection of the solution with the strongest competition. The major steps of the hybrid TOPSIS- EHO are outlined as follows:

1. Set randomly number of m populations to the elephant herd
2. Find the values to number of $\:n$ objective functions mentioned above and arrange these in a decision matrix D as given in Eq. (43).
  45 $$\:D=\:\left[\begin{array}{ccc}{f}_{11}\:\:\:\:\:\:{f}_{12}\:\:\:\:\:{f}_{13}&\:\cdots\:&\:{f}_{1n}\\\:⋮&\:\ddots\:&\:⋮\\\:{f}_{m1}\:\:\:{f}_{m2}\:\:\:{f}_{m3}&\:\cdots\:&\:{f}_{mn}\end{array}\right]$$
3. Apply the TOPSIS approach on matrix D and select a most suitable solution and its corresponding DGRs generation and consumer curtailed power based on the determined RCI values. Those who perform the best will either become leaders or the matriarch.
4. Update each clan’s elephant locations, except the worst and best
5. Update the best and worst elephant location in each group
6. Repeat steps 1–5 till reach the maximum number of iterations

The parameters used in the proposed MOEHO are as follow: the population size = 50, the maximum iteration count ($\:{T}_{max}$) = 100, $\:\alpha\:$ = 0.5, and $\:{\uprho\:}$ = 0.1. The proposed frame work has been simulated using MATLAB software and applied on IEEE 33 bus radial distribution network. The load flow calculation is applied using backward/forward method due to weakly meshed configuration of radial distribution system. The base power of the proposed distribution network is 100MVA with 12.66KV base voltage.

**Fig. 6:**
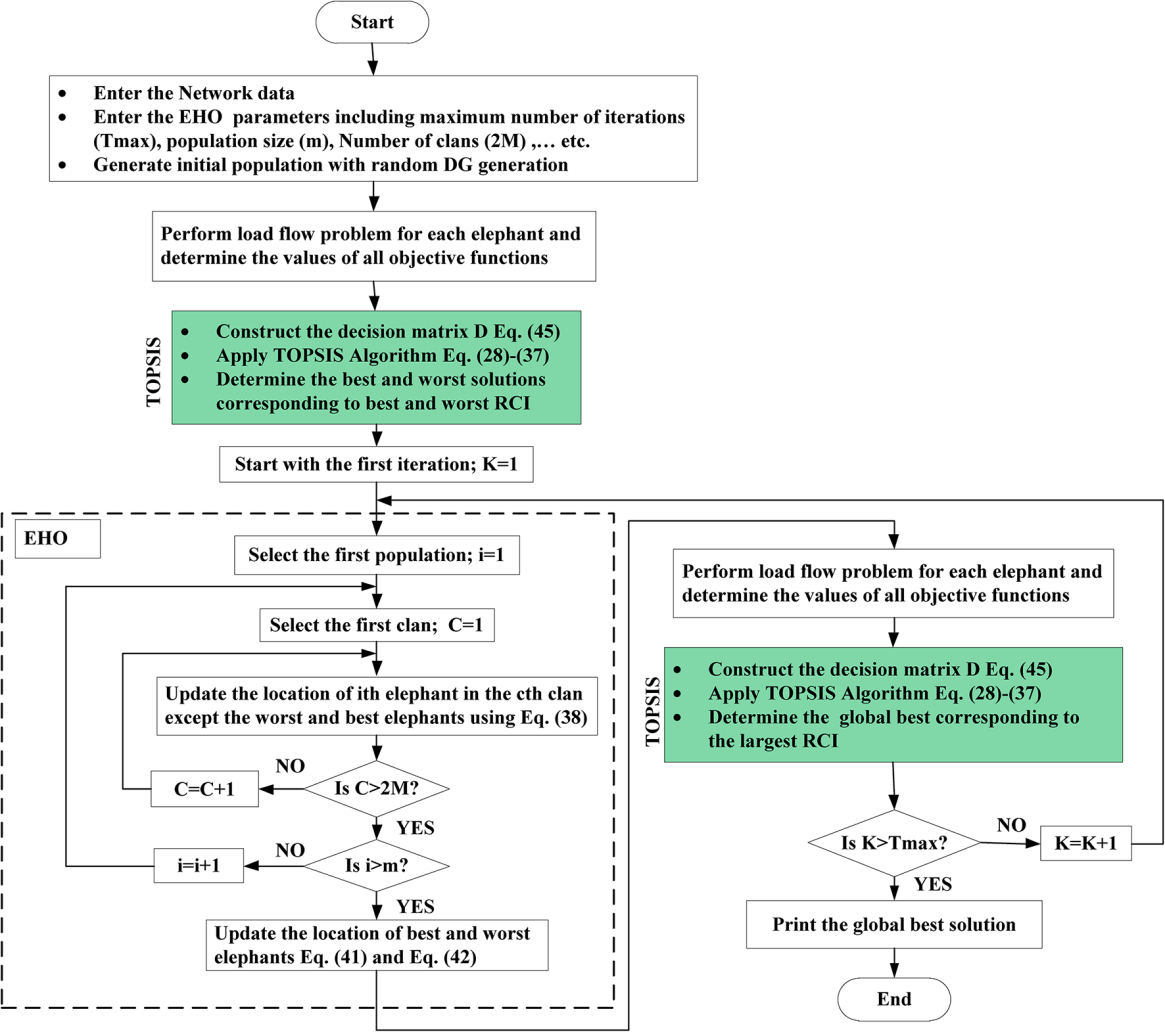
Flowchart of the TOPSIS- EHO.

Figure 6 Presents the proposed TOPSIS- EHO flowchart.

## Result and discussion

This section presents the outcomes and implications of applying the proposed strategy on the distribution network considering the two case studies of demand response programs (DRP based common incentive rate and DRP based individual incentive rate.

### Consumer’s optimal individual PNS at optimal common incentive rate

**Fig. 7:**
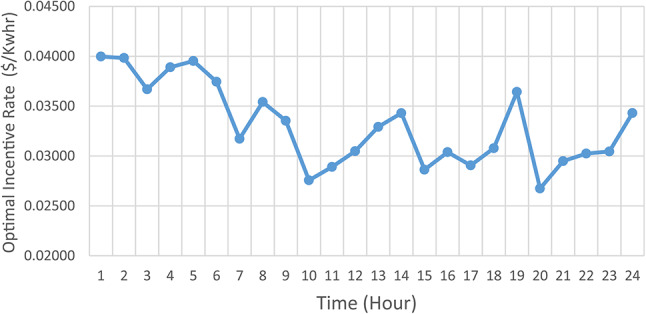
Real Time consumer’s optimal common Incentive rate.

**Fig. 8:**
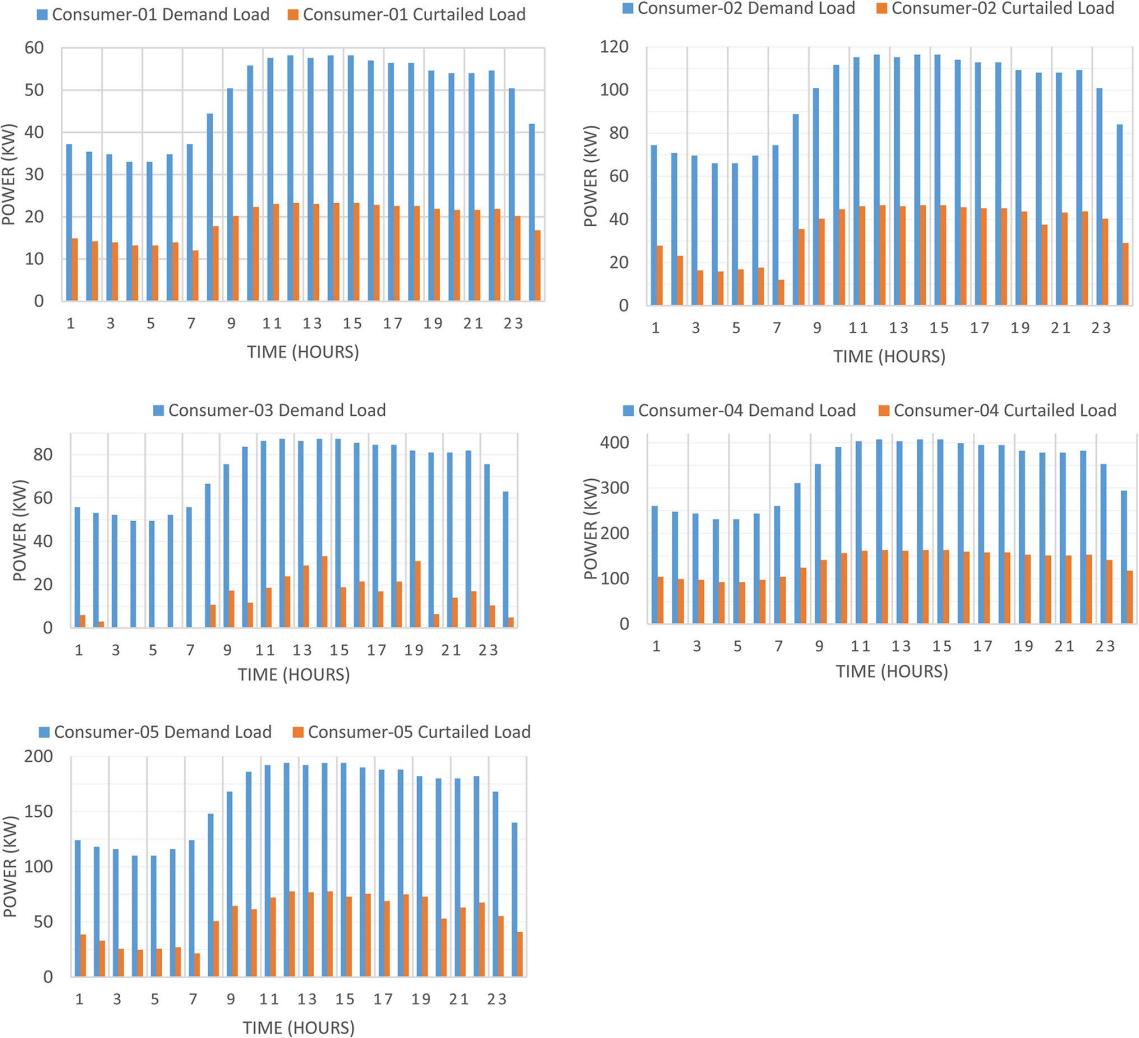
Demand load and curtailed load for consumers participate in DRP using CIR-DRP.

**Fig. 9:**
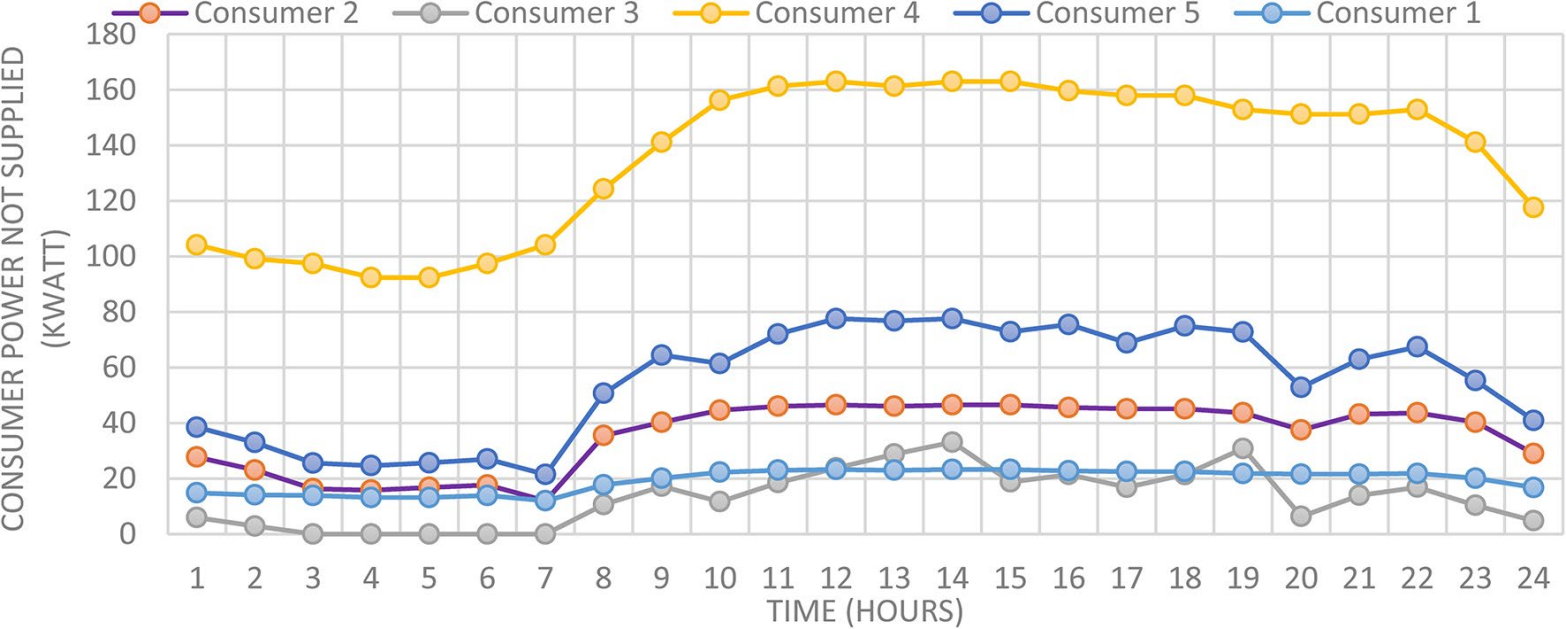
Consumer’s optimal individual Power Not Supplied using CIR-DRP.

**Fig. 10:**
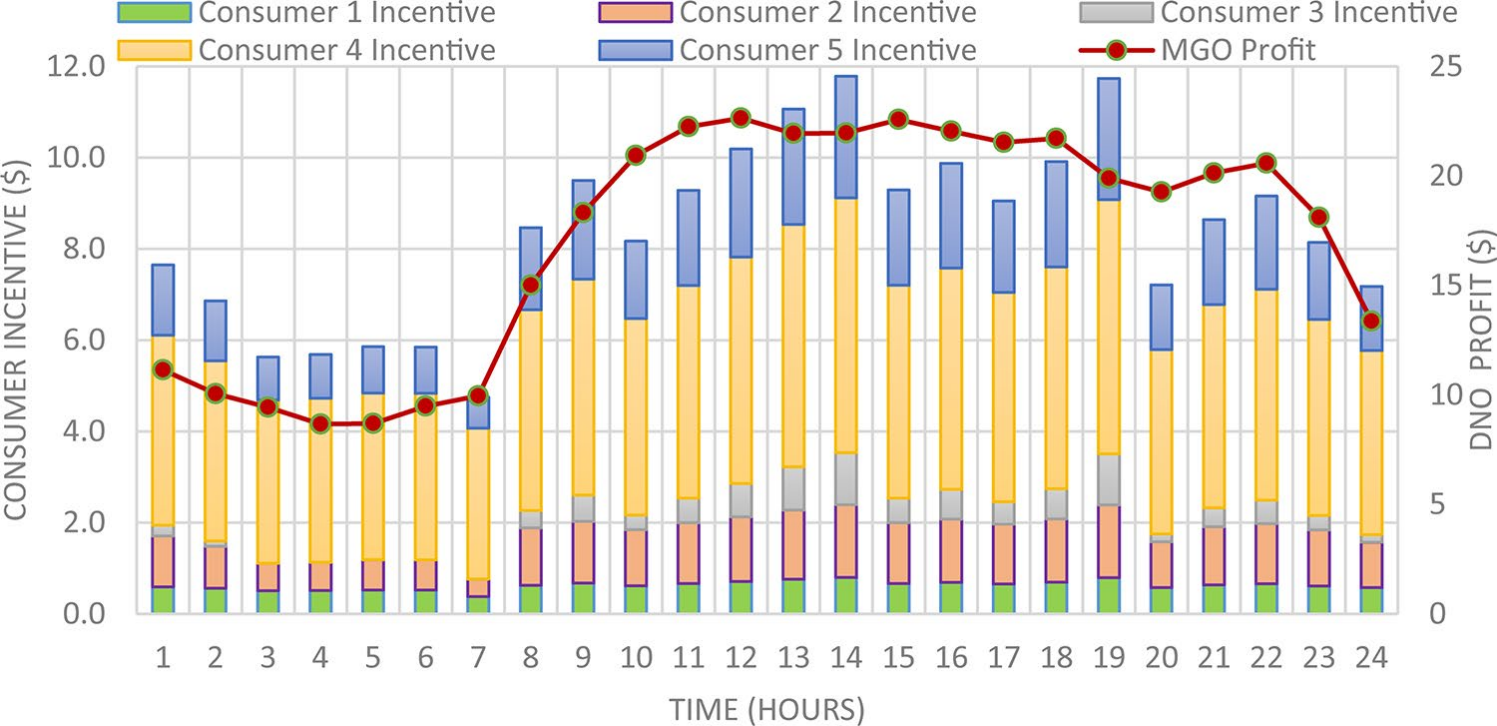
Total Incentive per hour with respect to DNO Profit using CIR-DRP.

Figure 7 presented optimal incentive rates throughout the day when the five consumers share the same incentive rate. It is clear, during the day the optimal incentive rate varies according to the loading condition. Figure 8 presents the Demand Load and Curtailed Load for each consumer involved in DRP due to the provided incentive rate. A comparison between the curtailed power from different consumers can be performed using Fig. 9. Even $$\:{C}_{5}$$ and $$\:{C}_{4}$$ have the same inability factor for power curtailment, however the power curtailment from consumer $$\:{C}_{4}$$ is higher than the power curtailment of consumer $$\:{C}_{5}$$ because $$\:{C}_{4}$$ has higher demand power as shown in Table 2. The same results are observed between consumer $$\:{C}_{2}$$ and $$\:{C}_{3}$$. On the other hand, even $$\:{C}_{1}$$ has less peak power compared to $$\:{C}_{3}$$, however in some intervals it has higher power curtailment, because $$\:{C}_{1}$$ has less inability factor compared to $$\:{C}_{3}$$ for power curtailment. The hourly total received incentive and DNO benefit are presented in Fig. 10. The achieved distribution network (DN) benefits are greater than the incentive amount received by all consumers during every interval. Table 5 provides an overview of the total energy not supplied for each consumer, along with the total incentive received by each consumer and the daily DNO profit. The daily DNO profit is 409.65 $/Day, while the daily incentive amount is 200.91 $/Day. consumer $$\:{C}_{4}$$ has the higher amount of energy curtailed during the day, hence consumer $$\:{C}_{4}$$ receives the highest incentive during the day.

**Table 5:** Consumer’s total PNS, incentive. Also, DNO Profit using CIR-DRP.

Consumer No.	Bus Location	Energy not supplied (kWh/day)	Percentage of Energy not supplied (%)	Received Incentives ($/day)	DNO Profit ($/day)
$\:{C}_{1}$	9	463.20	39.75	15.03	30.46
$\:{C}_{2}$	14	855.15	36.70	27.49	56.48
$\:{C}_{3}$	22	315.12	18.03	10.04	20.91
$\:{C}_{4}$	25	3262.56	40.00	105.85	214.54
$\:{C}_{5}$	30	1321.36	34.02	42.50	87.26
Total		6217.38	35.97	200.9	409.65

### Consumer’s optimal individual PNS at optimal individual incentive rate

**Fig. 11:**
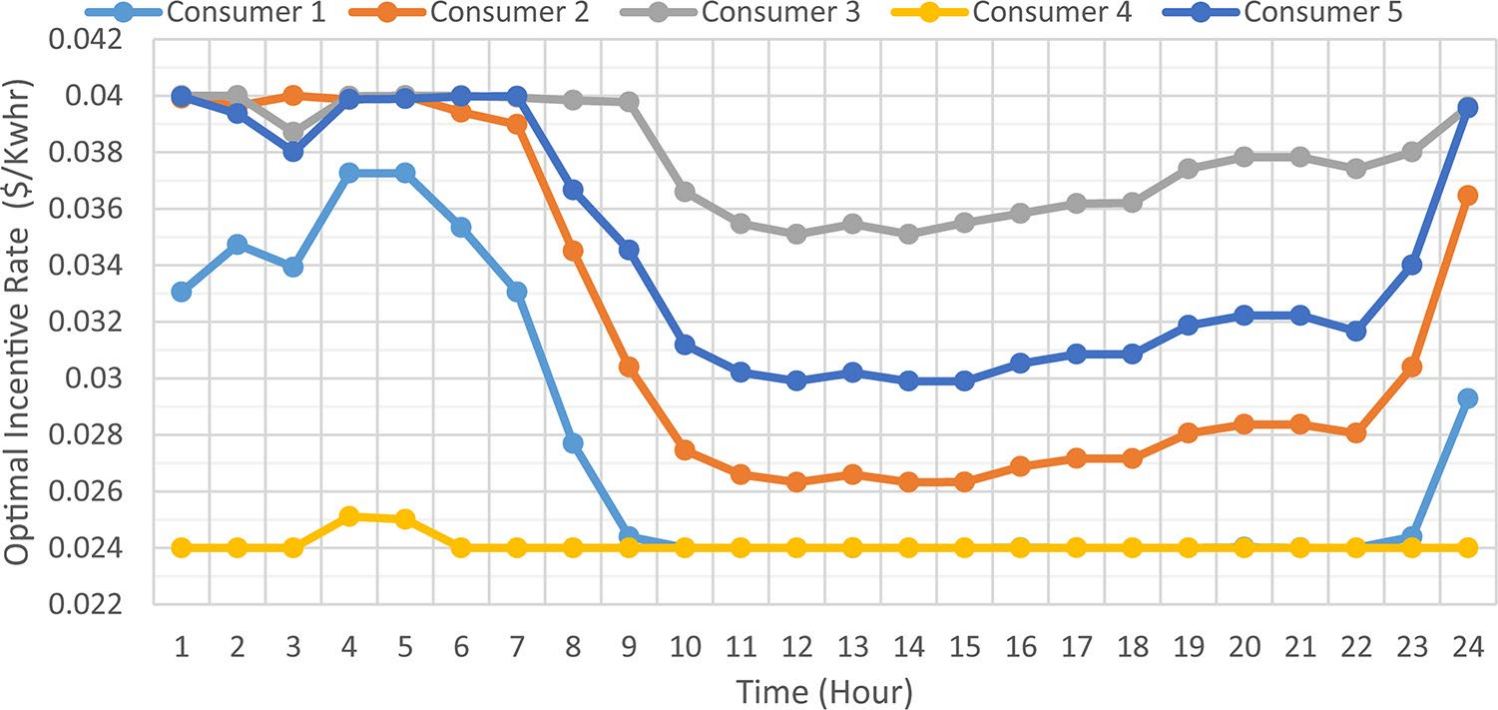
Hourly consumer’s optimal individual Incentive rate.

**Fig. 12:**
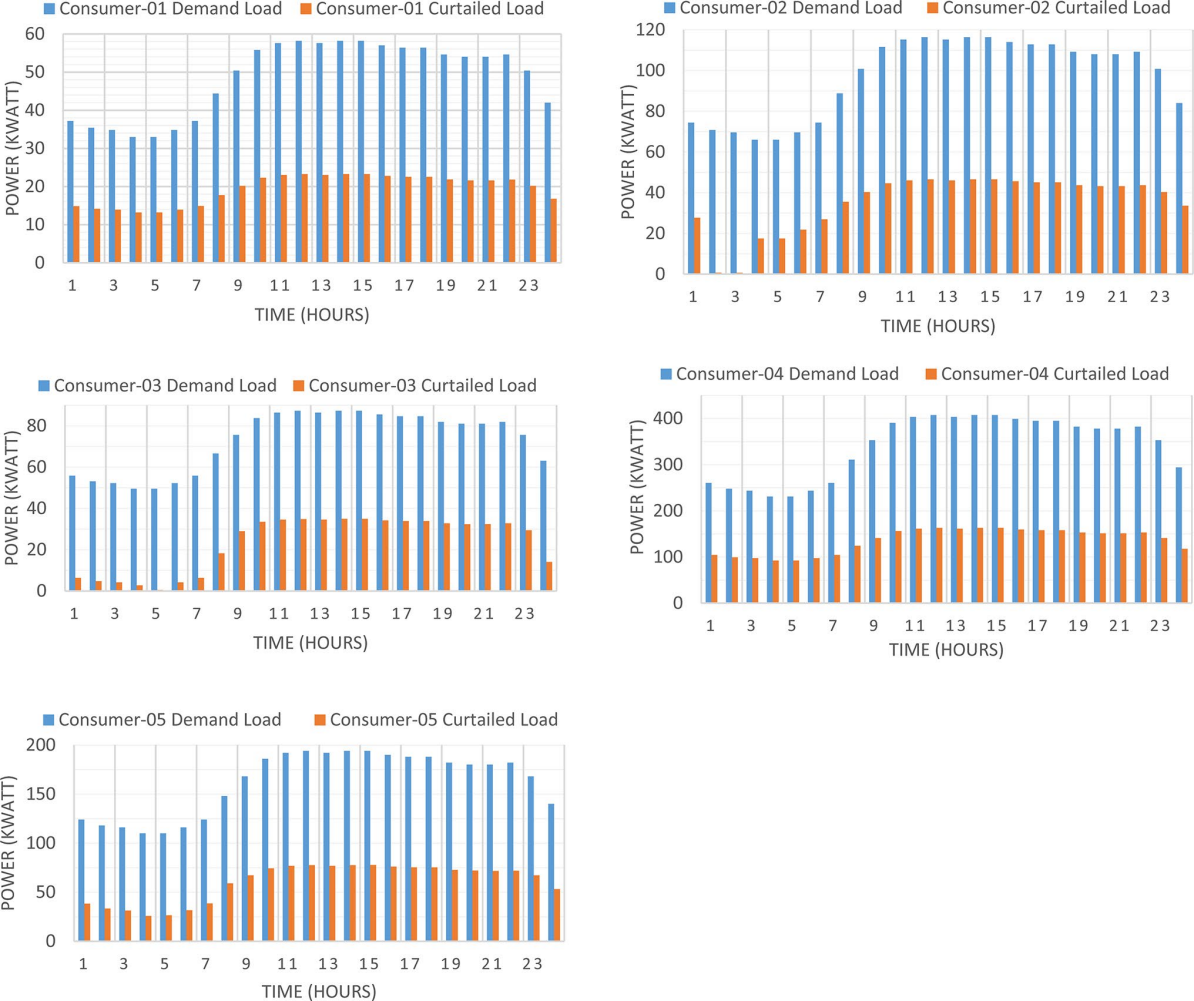
Demand load and curtailed load for every consumer participates in DRP using IIR-DRP.

**Fig. 13:**
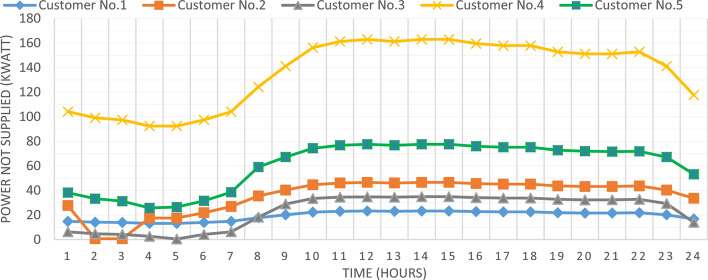
Optimal power not supplied to consumers participates in DRP using IIR-DRP.

**Fig. 14:**
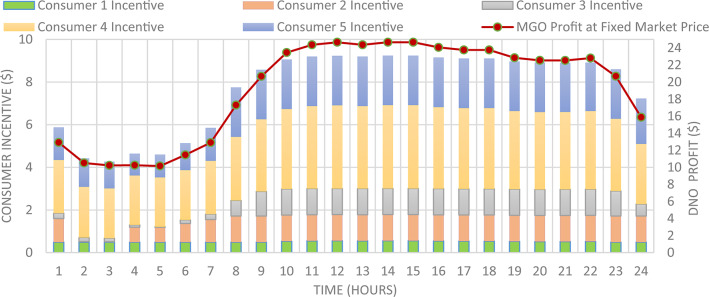
Total Incentive per hour with respect to DNO Profit using IIR-DRP.

The DNO profit is enhanced by assignee different incentive rates for consumes participating in the DRP as presented in Fig. 11. It is clear, the optimal incentive rate varies from consumer to consumer as well as varies along the day. The incentive rate for the same consumer declines with increase in consumer demand. The original demand and power curtailment for different consumers are illustrated in Fig. 12. The amount of power not supplied for each consumer along the day is illustrated in Fig. 13. For the same consumer, the amount of power curtailed from each consumer along the day is proportional to the consumer demand which makes positive effect to reduce the DN loading during peaks. consumer $$\:{C}_{4}$$ provides more power curtailment compared to $$\:{C}_{5}$$ even they have the same inability factor, because $$\:{C}_{4}$$ has higher demand compared to $$\:{C}_{5}$$. Also, The same with consumers $$\:{C}_{2}$$ and $$\:{C}_{3}$$. The DNO profit at every hour along the day is greater than the incentive paid for consumers as presented in Fig. 14. The total incentive to consumers is 185.12 $/day while the total DNO profit is 461.39 $/day as shown in Table 6.

**Table 6:** Consumers total PNS, incentive and DNO profit using IIR-DRP.

Consumer	Bus location	ENS (kWh/day)	Percentage of ENS (%/day)	Received incentive ($/day)	DNO profit ($/day)
C 1	9	466.06	40.00	12.44	33.32
C 2	14	848.84	36.42	25.33	58.03
C 3	22	559	31.99	20.70	34.19
C 4	25	3262.55	40.00	78.51	241.88
C 5	30	1447.20	37.26	48.14	93.97
Total		6583.65	38.09	185.12	461.39

**Fig. 15:**
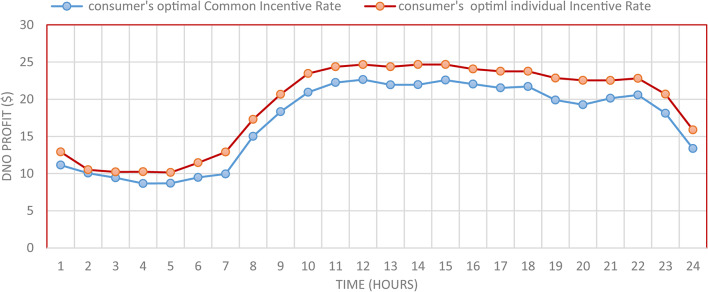
DNO Profit for every two study cases.

The individual incentive rate for each consumer provides better DNO along every hour as well as along the day compared to common incentive rate as illustrated in Fig. 15. The total DNO profit with individual incentive rate is 461.39 $/day, while it is 409.65 $/day using common incentive rate, which mean enhancement by 12.63% as shown in Table 7. This profit enhancement is associated with consumers incentive reduction from 200.9 $/day to 185.12 $/day. Moreover, the demand reduction of these consumers is increased from 6217.38 kwh/day to 6583.65 kwh/day.

**Table 7:** Summary of total ENS, incentive and DNO profit with different incentive strategies.

Study case	Total ENS (kWh/day)	Total Percentage of ENS (%/day)	Incentives received ($/day)	DNO Profit ($/day)
Common incentive rate	6217.38	35.97	200.90	409.65
Individual incentive rate	6583.65	38.09	185.12	461.39

### Multi-objective optimal power sharing without DRP

**Fig. 16:**
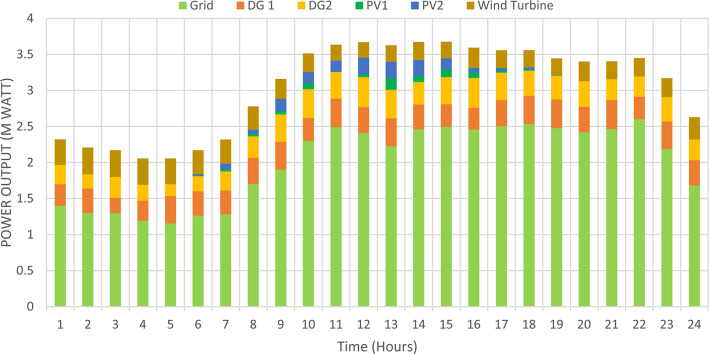
Shared power between DGRs and grid before implementing load demand response program.

**Fig. 17:**
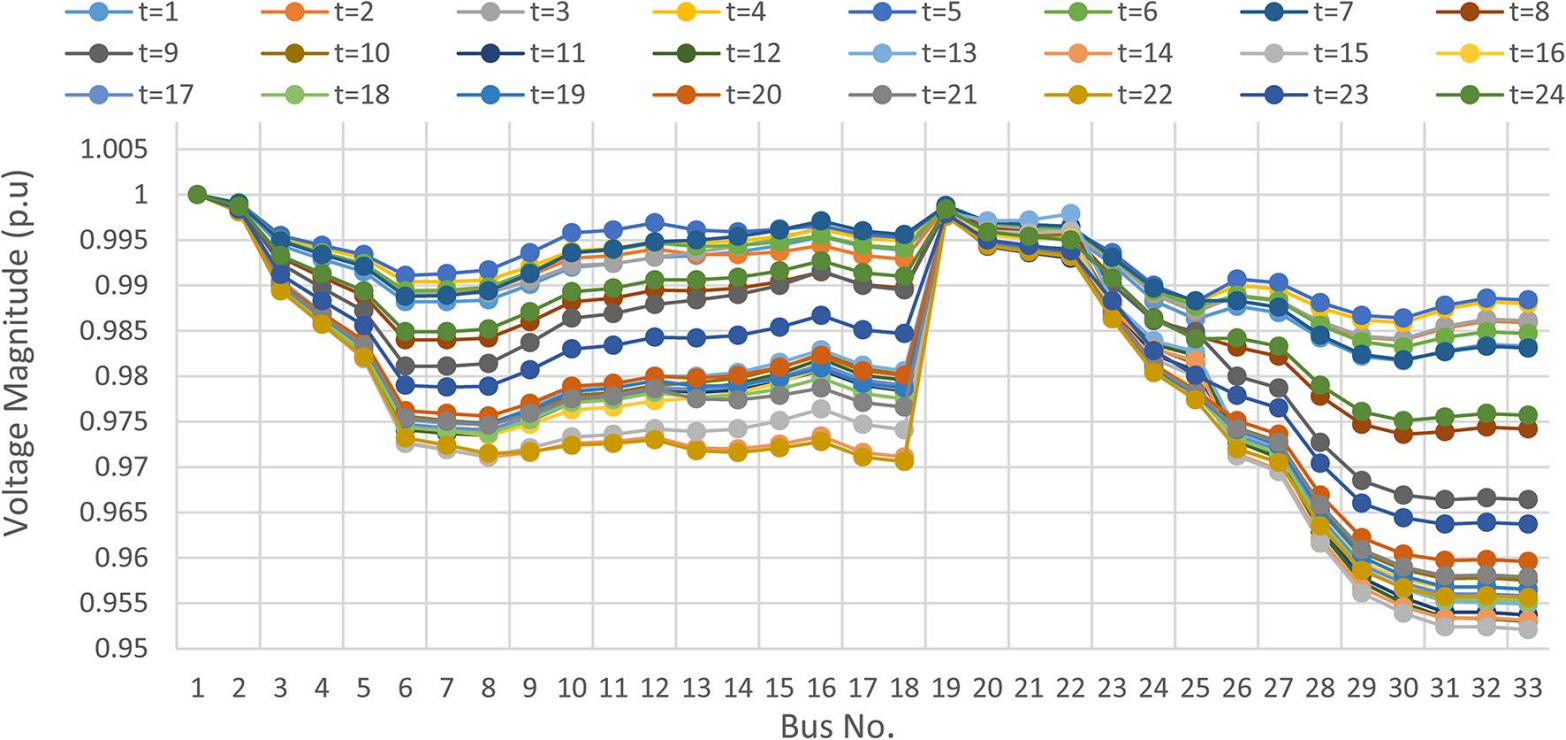
Hourly voltage profile at every bus without considering DRP.

Herein, the proposed TOPSIS and EHO are used to find the optimal shared power between DGRs and the grid without implementing DRP as obtained in Fig. 16. The proposed TOPSIS and EHO methodology is able to maintain the voltage at all buses within permissible limits (above 95%) with variability of load and renewable generations as illustrated in Fig. 17. The maximum value of total voltage deviation is 0.0273 p.u and the worst voltage stability margin of 0.8161 pu, total energy loss along the day of 1.085 MWh/day, and the total generation cost of 6798.41 $/day. This optimal power sharing and local generation improve the voltage at every bus, the total power losses, the generation cost and the stability margins, however further enhancement is observed by applying the DRP as will be discussed later.

### Multi-objectives optimal power sharing with CIR-DRP

**Fig. 18:**
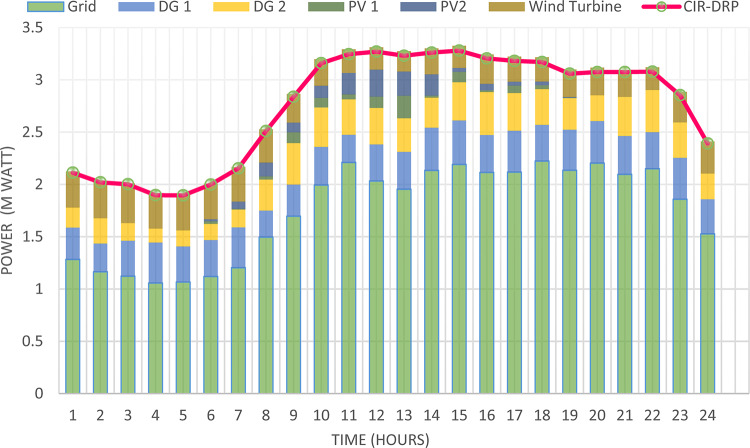
Power outputs of DGRs and grid with respect to total demand load after implementing DRP.

**Fig. 19:**
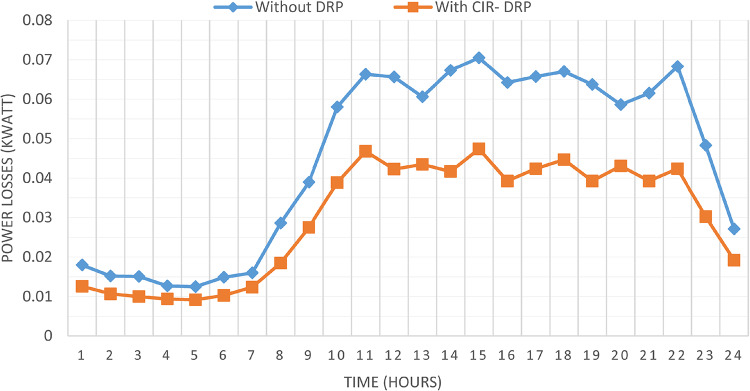
CIR-DRP impact on total power losses.

**Fig. 20:**
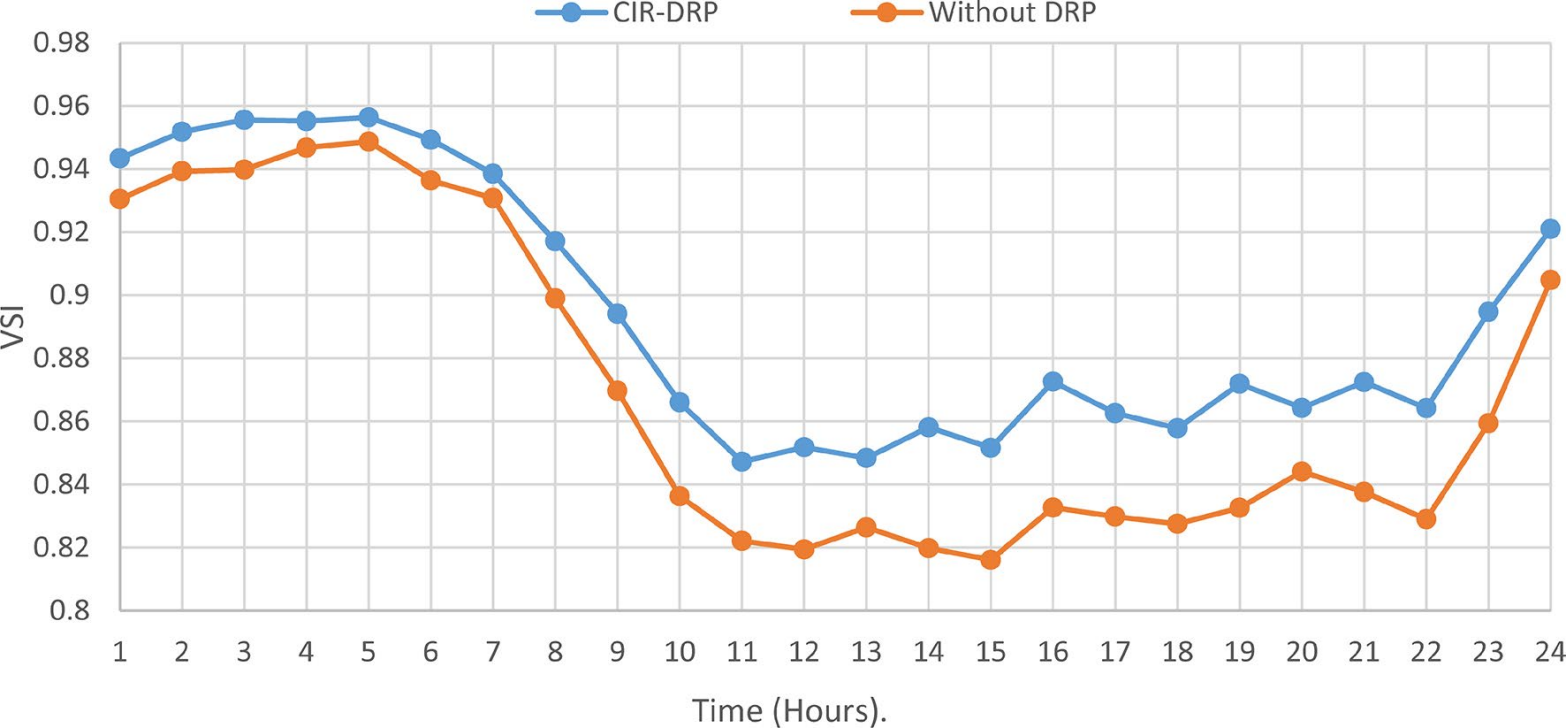
CIR-DRP Impact on VSI.

**Fig. 21:**
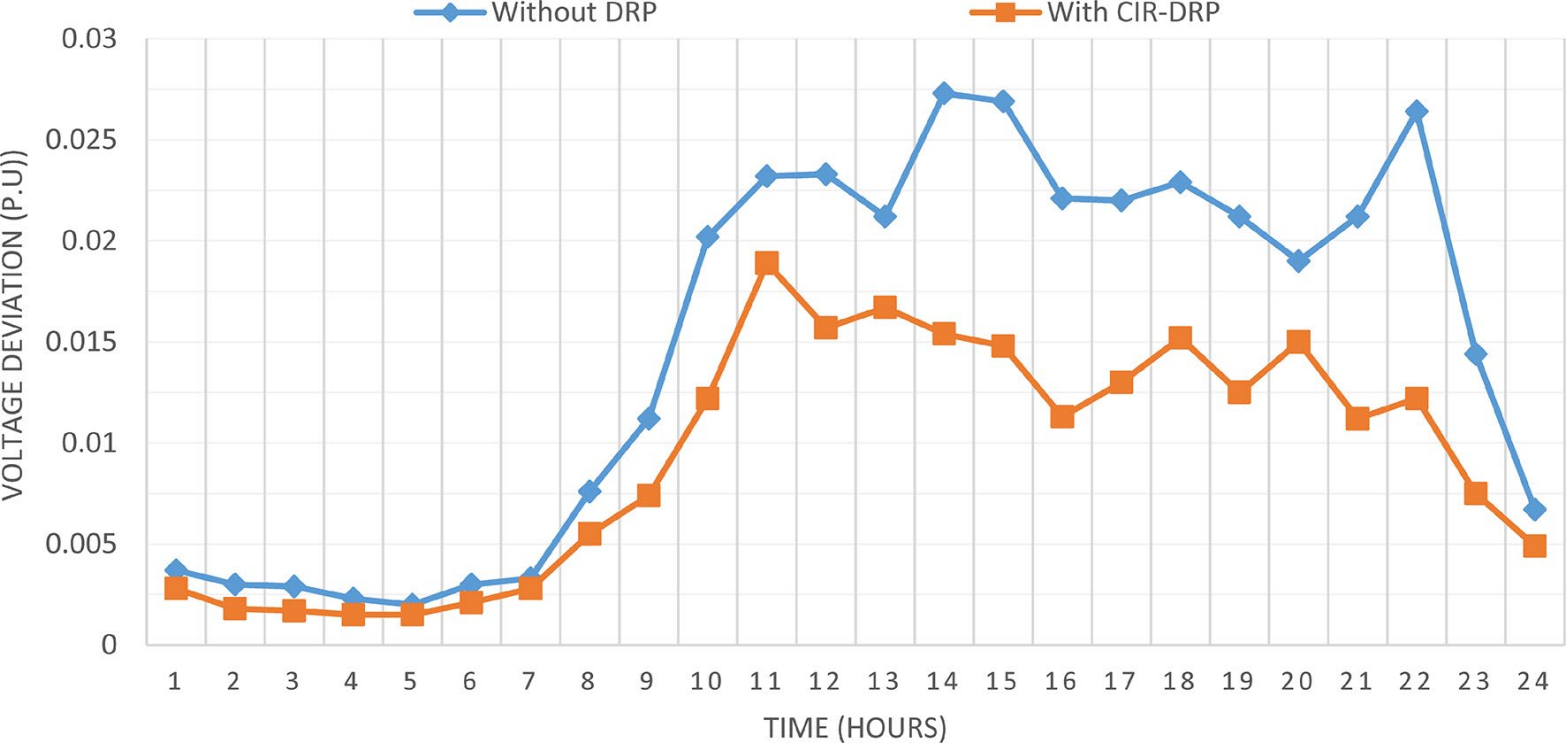
CIR-DRP impact on the total voltage deviation.

**Fig. 22:**
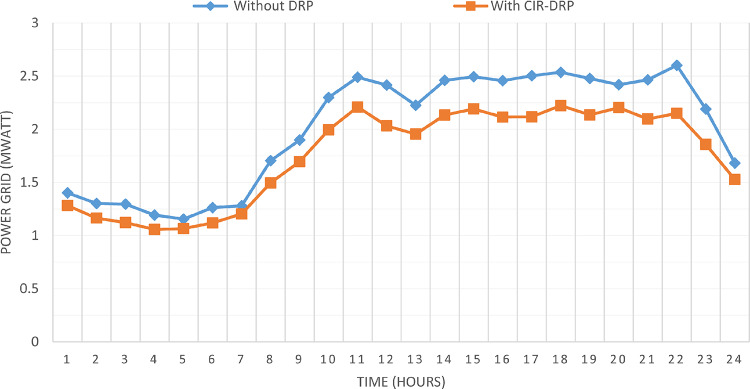
CIR-DRP Impact on exchange with the grid.

**Fig. 23:**
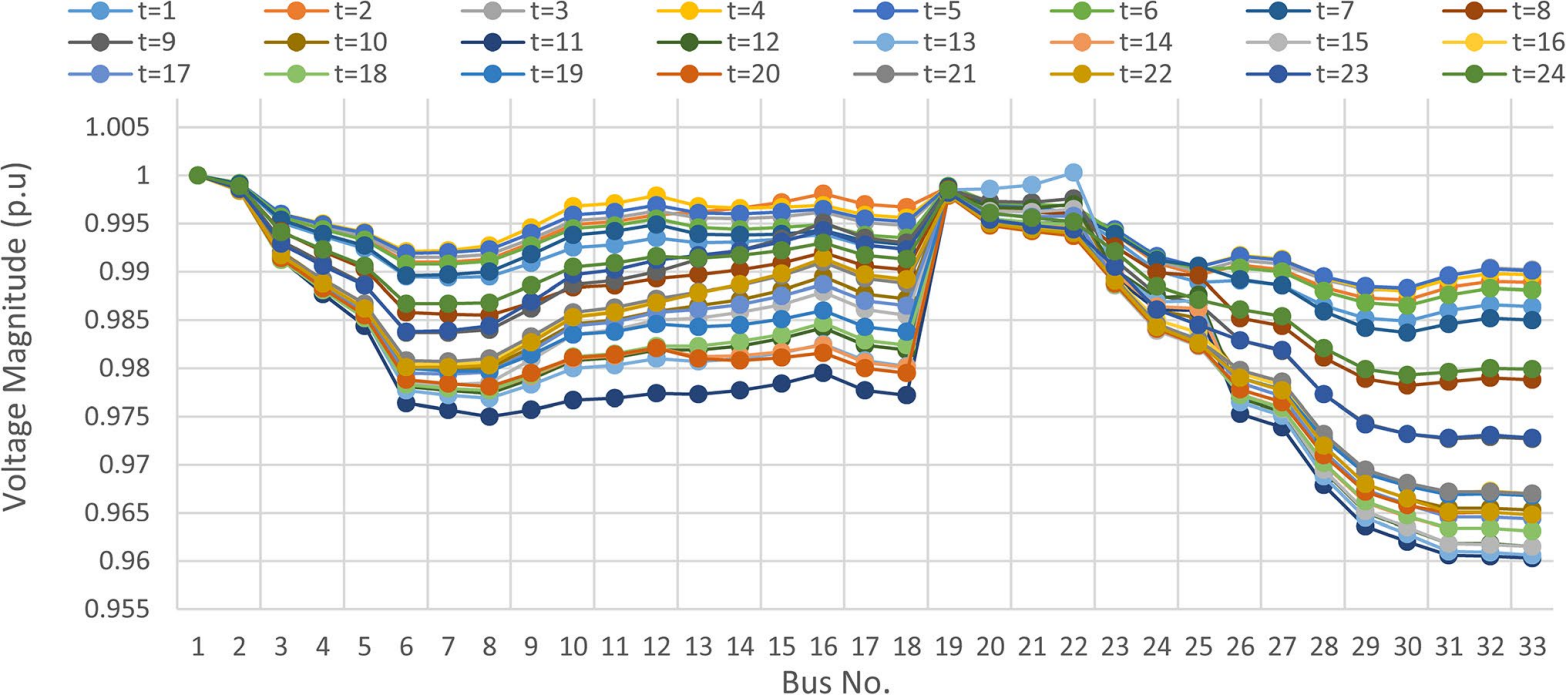
Hourly voltage profile at every bus considering CIR-DRP.

The proposed TOPSIS and EHO technique is utilized to achieve multi-objective optimal shared power between Distributed Generation Resources (DGRs) and the grid at optimal common Incentive Rate for all consumers participating in DRP as depicted in Fig. 18. Due to reduction of the loading at the presence of the DRP, the total power losses at every hour are reduced as illustrated in Fig. 19. The total energy losses per day is reduced to 0.7211 MWh/day compared to 1.085 MWh/day before implementing the DRP. The voltage stability margin is enhanced along the day as illustrated in Fig. 20. Where the minimum VSI is enhanced to 0.8472 compared to 0.8161 without DRP. Additionally, the total voltage deviation along the distribution network is enhanced using DRP as presented in Fig. 21, where the maximum TVD is 0.0189 compared to 0.0273 without DRP. Moreover, the DRP decreases the energy received from the utility grid along the day as shown in Fig. 22 with a total daily energy purchased of 42.14 MWh/day with 4138.25 $/day. Eventually, the voltage profile along the day is kept with permissible limit as illustrated in Fig. 23 with enhancement in the minimum voltage to 0.9603 compared to 0.9521 without DRP.

### Multi-objectives optimal power sharing with IIR-DRP

**Fig. 24:**
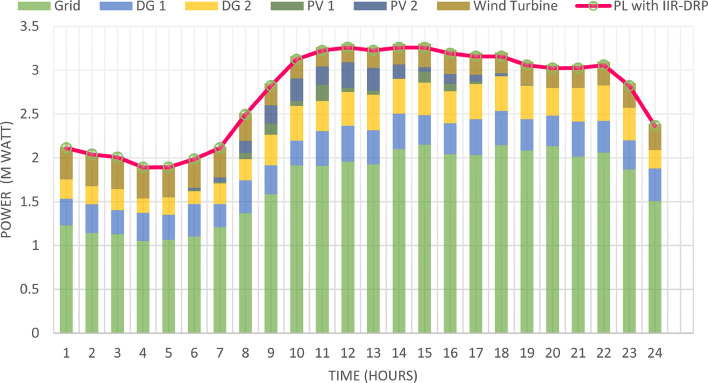
Power outputs of DGRs and grid with respect to total demand load after implementing IIR-DRP.

**Fig. 25:**
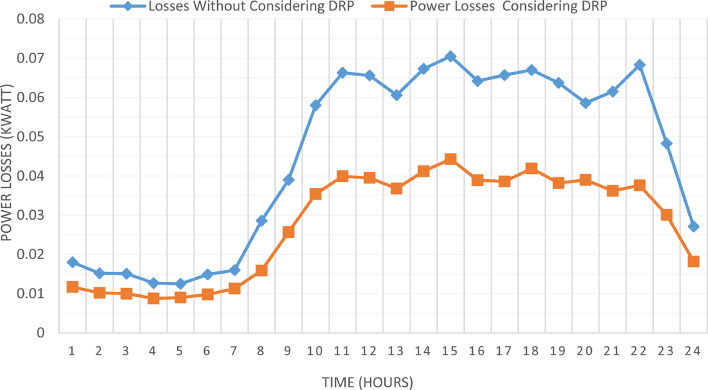
IIR-DRP impact on total power losses.

**Fig. 26:**
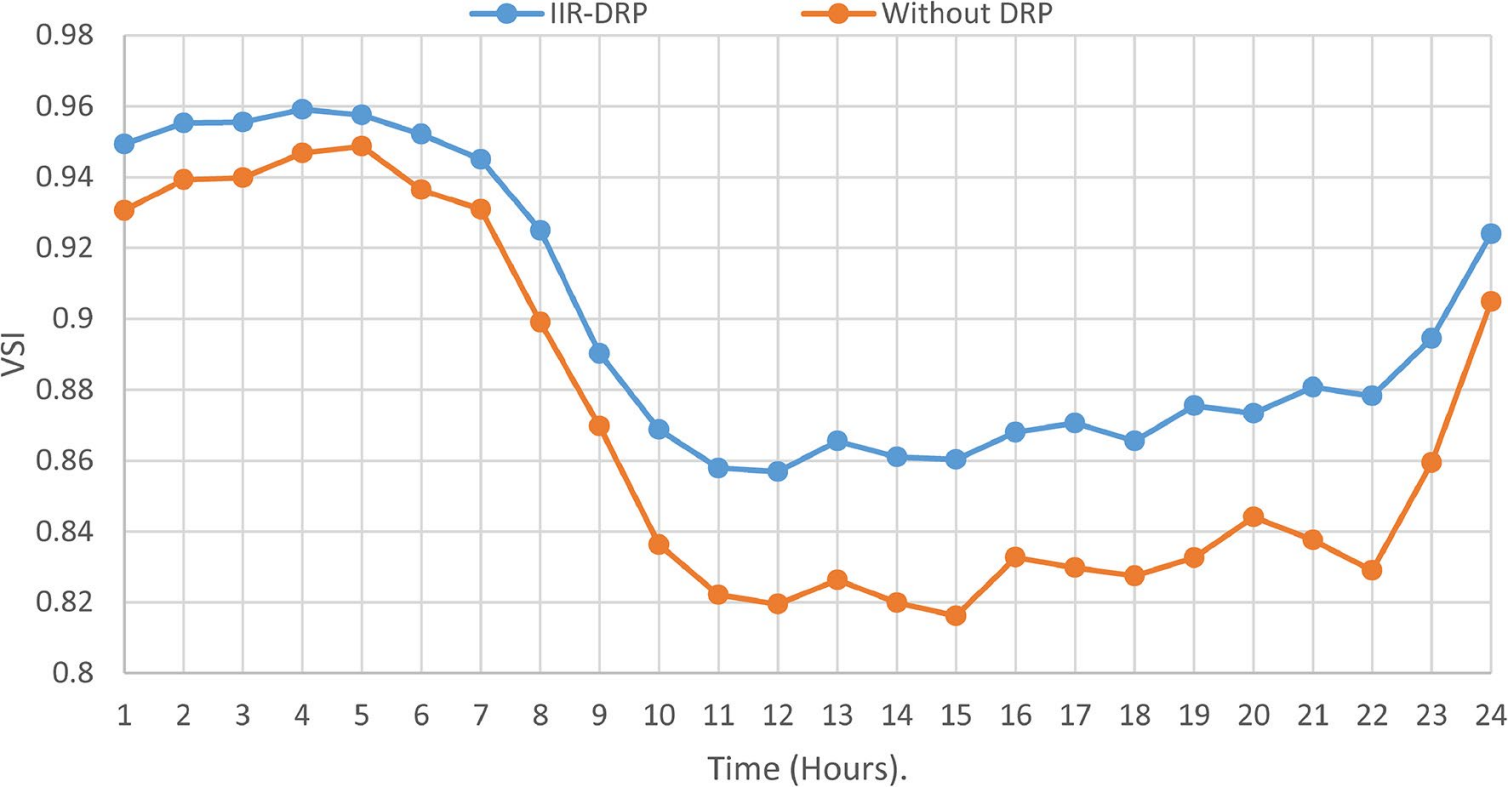
IIR-DRP impact on VSI.

**Fig. 27:**
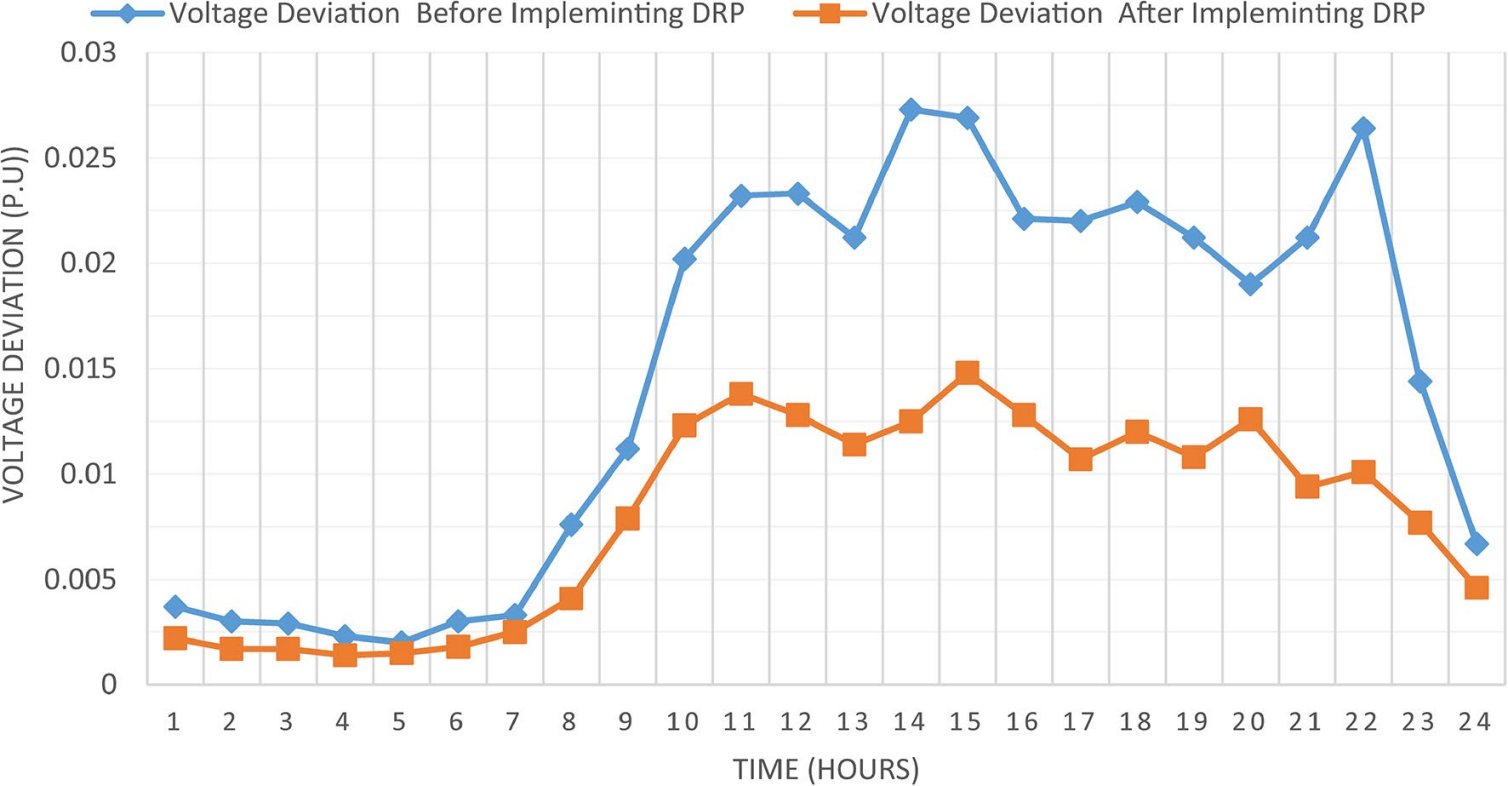
IIR-DRP impact on voltage deviation.

**Fig. 28:**
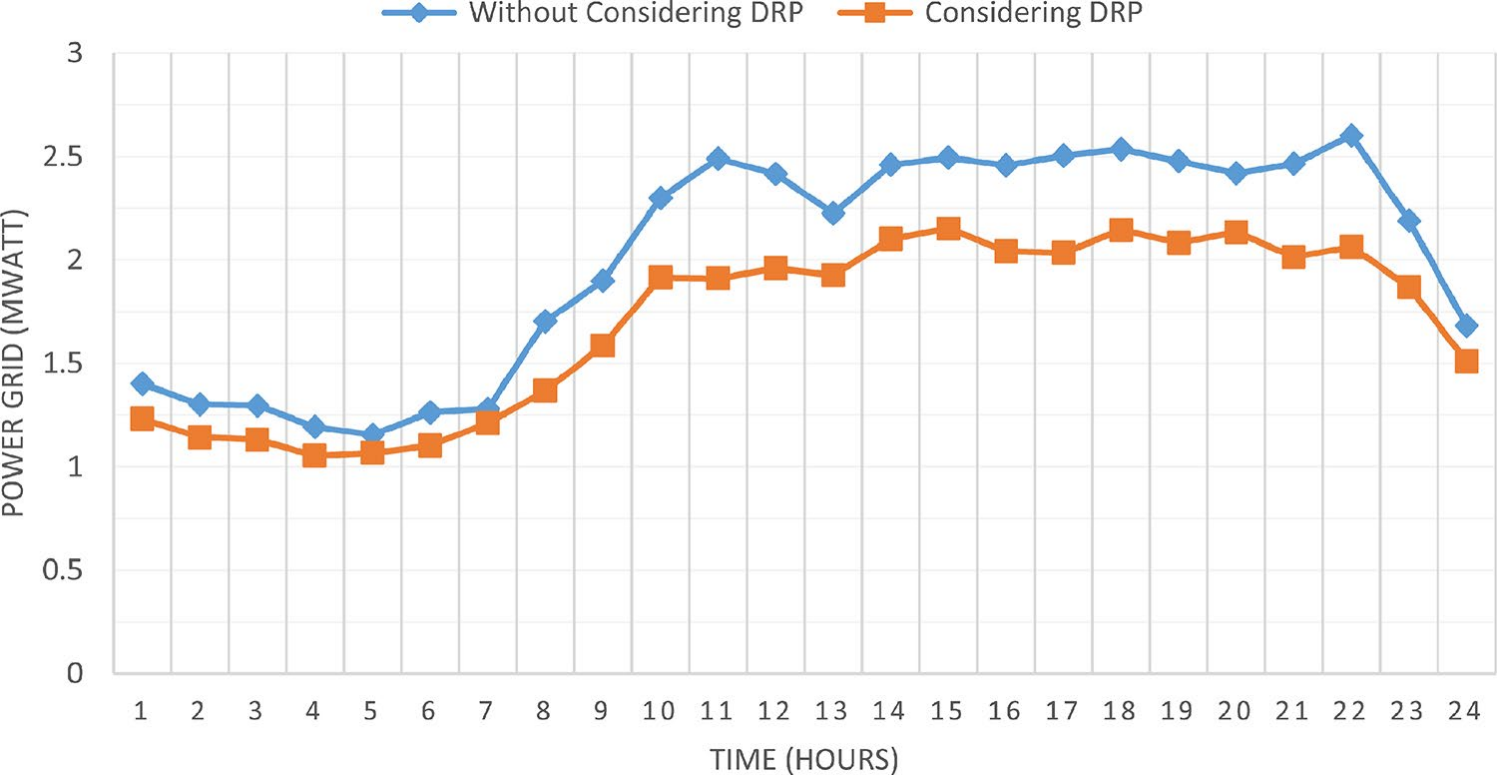
IIR-DRP impact on exchange with the grid.

**Fig. 29:**
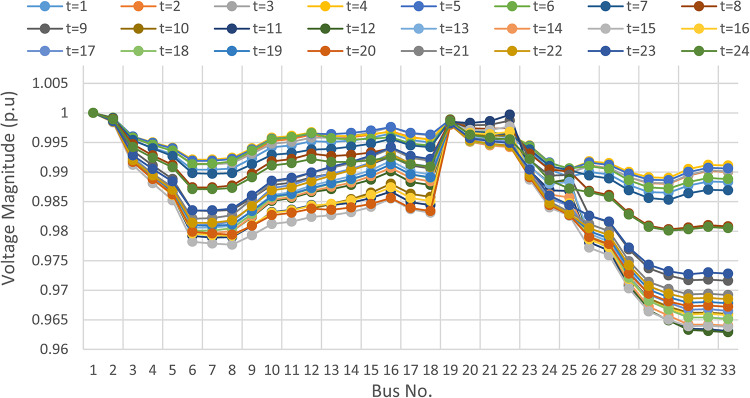
Hourly voltage profile at every bus considering IIR-DRP.

The power sharing among the grid and different generation resources within the network is optimized considering the load shaping after implementing IIR-DRP as illustrated in Fig. 24. The IIR-DRP reduces the power losses within the network at every hour compared to the operation without DRP as presented in Fig. 25 with total energy losses of 0.6682 MWh/day compared to 1.085 MWh/day without DRP. In addition, the network voltage stability is improved as illustrated in Fig. 26, since the minimum VSI is enhanced to 0.8569 compared to 0.8161 without DRP. Furthermore, the IIR-DRP enhances the voltage deviation at every hour on the day as shown in Fig. 27, where the maximum TVD is improved to 0.0148 compared with 0.0273 without DRP. Moreover, the IIR-DRP reduces the required power from the grid along the day as shown in Fig. 28. Eventually, the voltage profile within the network is enhanced using IIR-DRP as illustrated in Fig. 29, where the minimum node voltage is 0.9629 pu. The total cost using IIR-DRP is 7285.495 $/day which is less than that without DRP.

**Fig. 30:**
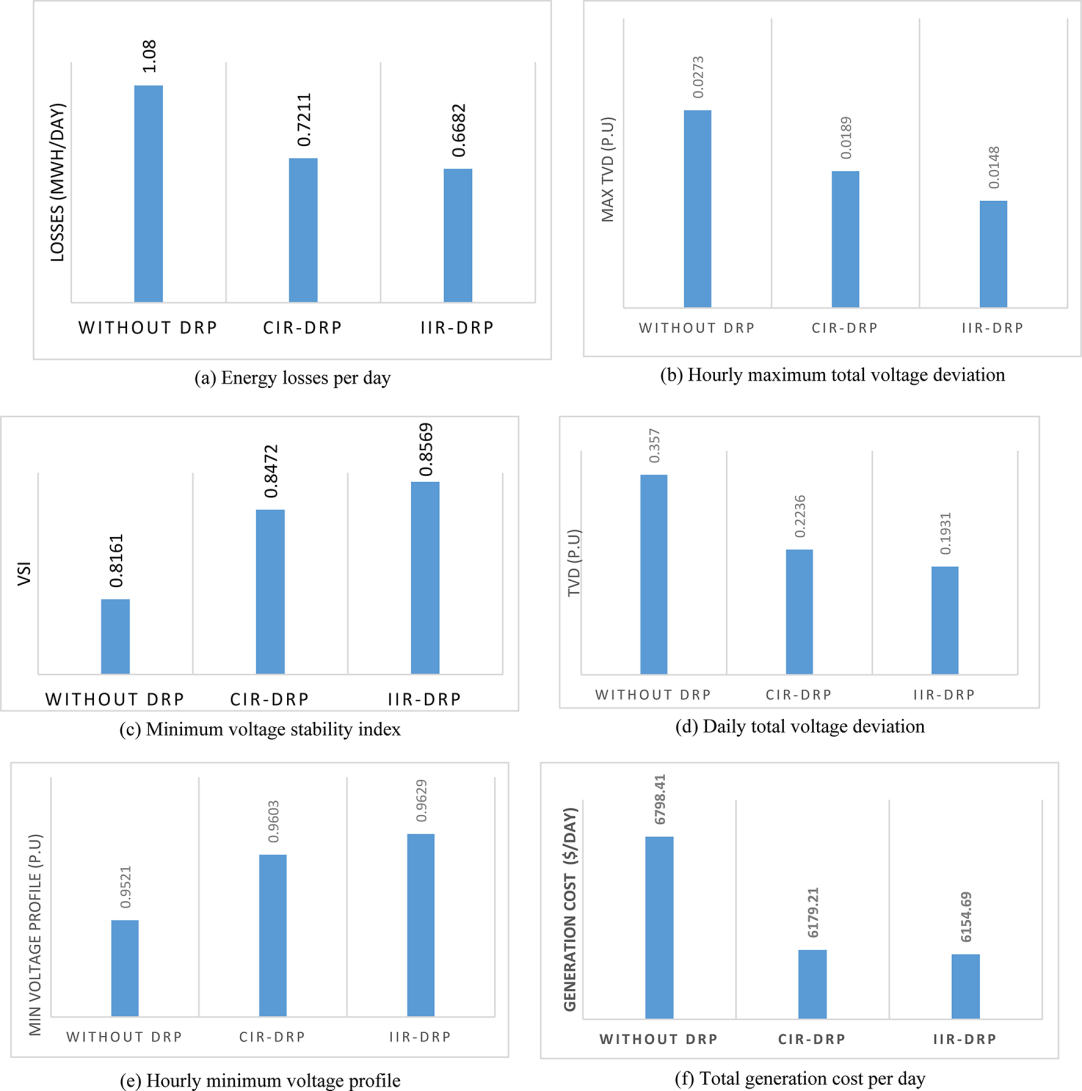
Comparison between operation without DRP, with CIR-DRP, and IIR-DRP.

Implementing demand response program with optimal power sharing leads to significant improvements in various performance metrics. It results in a substantial reduction in total energy losses per day as illustrated in Fig. 30a, since the total losses is reduced from 1.0847 MWh/day to 0.7211 MWh/day and from 1.0847 MWh/day to 0.6682 MWh/day using CIR-DRP and IIR-DRP respectively. Thereby, the proposed framework improves the overall efficiency. In addition to reduction in hourly maximum value of voltage deviation from 0.0273 p.u to 0.0189 p.u and from 0.0273 p.u to 0.0148 p.u using CIR-DRP and IIR-DRP respectively as illustrated in Fig. 30b. In addition, with DRP the minimum value of voltage stability index of the network throughout the day is enhanced from 0.8161 to 0.8472 and from 0.8161 to 0.8569 using CIR-DRP and IIR-DRP as illustrated in Fig. 30c. Moreover, the daily total voltage deviation along the network is enhanced from 0.357 p.u to 0.2236 p.u and from 0.357 p.u to 0.1931 p.u using CIR-DRP and IIR-DRP respectively as presented in Fig. 30d. Also, the minimum voltage profile throughout the day increased from 0.9521 p.u before implementing DRP to 0.9603 p.u in case of CIR-DRP and 0.9629 p.u in case of IIR-DRP as shown in Fig. 30e. Eventually, the proposed framework reduces the total generation cost from 6798.41 $/day to 6179.21 $/day and from 6798.41 $/day to 6154.69 $/day using CIR-DRP and IIR-DRP respectively as presented in Fig. 30f. Table 8 summarized the technical and economic benefits in addition to the environmental benefits after applying the proposed strategy to each case study.

**Table 8:** Benefits after applying the proposed strategy for every case study.

Case study	Daily total energy losses (MWh/day)	Daily total voltage deviation (*p*.u)	Daily total gas emissions limitation ($/day)	Daily min voltage profile (*p*.u)	Daily minimum VSI (*p*.u)	Daily total operational cost ($/day)	Daily total power grid (MWh/day)
Before DRP	1.08	0.357	1201.1364	0.9521	0.8161	6798.411	48.2064
DRP based CIR	0.7211	0.2236	1165.4705	0.9603	0.8472	6179.2091	42.141
DRP based IIR	0.6682	0.1931	1130.8069	0.9629	0.8569	6082.69	40.7495

**Fig. 31:**
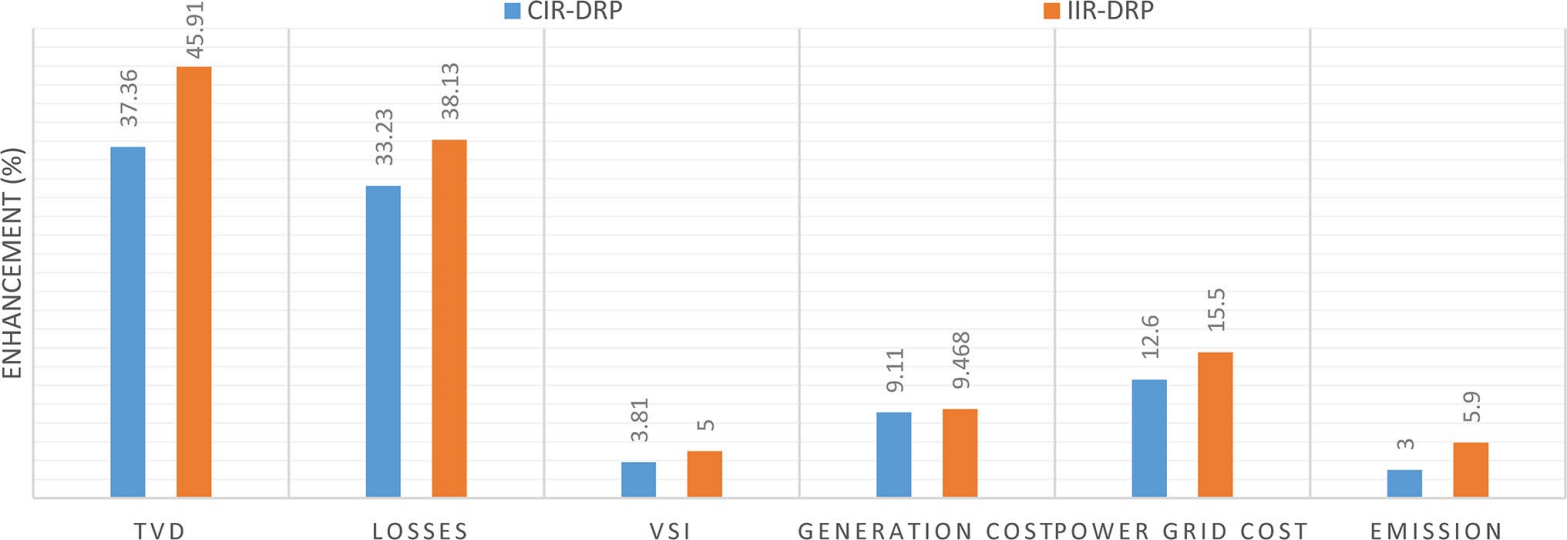
Enhancement in different indices.

**Fig. 32:**
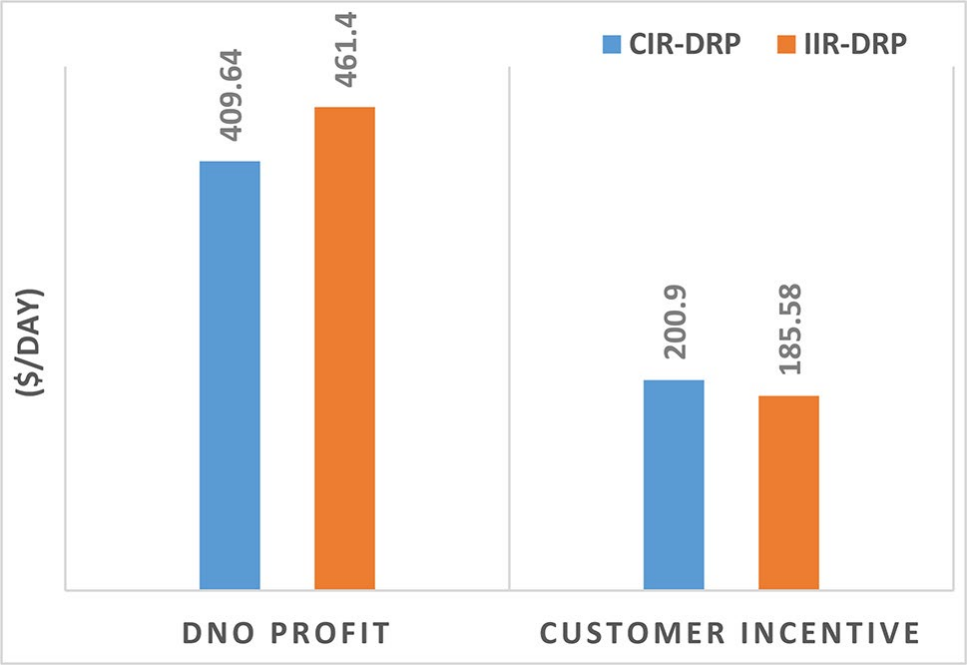
DNO Profit and consumer incentive.

The percentage of enhancement in different meters is provided in Fig. 31. It is clear, the DRP provides enhancement in all system metrics. Furthermore IIR-DRP provide better enhancement compared to CIR-DRP. In addition, the IIR-DRP provides more benefit to the DNO at less incentive cost for the consumers as illustrated in Fig. 32.

## Conclusion

This study introduces a contemporary approach for distribution network operator (DNO) to effectively manage modern distribution networks and facilitate coordination among consumers participating in demand response program (DRP). The proposed framework involves two stages. In the first stage, the curtailed power for consumers participating in DRP is managed and controlled to optimize the DNO profit by adjusting the applied incentive rate considering the consumers discomfort due to this power curtailment. The second stage involves the multi-objective optimal power sharing between DGRs and the grid to reduce total power losses, reduce voltage deviation, maximize voltage stability index, and minimize operational and maintenance costs, as well as the gasses emission limitation. Elephant Herding Optimization technique (EHO) with TOPSIS is applied to solve multi-objective problem.

The applied DRP is an effective way to provide technical and economic benefits for the grid operators such as reducing the total energy losses by 38.13%, reducing the maximum deviation in the nod voltage by 45.78%, enhance the voltage stability to 5%, reducing the required grid power by 15.5% and reducing the total generation cost by 9.468%. These enhancements are associated with DNO profit and consumers incentives. Individual incentive rate for each consumer achieves better value for DNO profit in addition reduces the consumer’s incentive. Furthermore, the effect of the individual incentive rate extended to reduce the real power losses, reduces the generation cost, reduces the voltage deviation, enhances the voltage stability index, and reduces the required power received from the grid compared to common incentive rate for all consumers. Our future work will be involved a comparison between the proposed algorithm and other algorithms to ensure its effectiveness. Also, applying the proposed framework on larger distribution systems with a large number of consumers participate in DRP.

## Data Availability

All data generated or analyzed during this study are included in this published article.
